# The calcitonin-like system is an ancient regulatory system of biomineralization

**DOI:** 10.1038/s41598-020-64118-w

**Published:** 2020-05-05

**Authors:** João C. R. Cardoso, Rute C. Félix, Vinícius Ferreira, MaoXiao Peng, Xushuai Zhang, Deborah M. Power

**Affiliations:** 1 Comparative Endocrinology and Integrative Biology, Centre of Marine Sciences, Universidade do Algarve, Campus de Gambelas, 8005-139 Faro, Portugal; 2 International Research Center for Marine Biosciences, Ministry of Science and Technology, Shanghai Ocean University, Shanghai, China; 3 Key Laboratory of Exploration and Utilization of Aquatic Genetic Resources, Ministry of Education, Shanghai Ocean University, Shanghai, China

**Keywords:** Computational biology and bioinformatics, Evolution, Physiology

## Abstract

Biomineralization is the process by which living organisms acquired the capacity to accumulate minerals in tissues. Shells are the biomineralized exoskeleton of marine molluscs produced by the mantle but factors that regulate mantle shell building are still enigmatic. This study sought to identify candidate regulatory factors of molluscan shell mineralization and targeted family B G-protein coupled receptors (GPCRs) and ligands that include calcium regulatory factors in vertebrates, such as calcitonin (CALC). In molluscs, CALC receptor (CALCR) number was variable and arose through lineage and species-specific duplications. The Mediterranean mussel (*Mytilus galloprovincialis)* mantle transcriptome expresses six CALCR-like and two CALC-precursors encoding four putative mature peptides. Mussel CALCR-like are activated *in vitro* by vertebrate CALC but only receptor CALCRIIc is activated by the mussel CALCIIa peptide (EC_50_ = 2.6 ×10^−5^ M). *Ex-vivo* incubations of mantle edge tissue and mantle cells with CALCIIa revealed they accumulated significantly more calcium than untreated tissue and cells. Mussel CALCIIa also significantly decreased mantle acid phosphatase activity, which is associated with shell remodelling. Our data indicate the CALC-like system as candidate regulatory factors of shell mineralization. The identification of the CALC system from molluscs to vertebrates suggests it is an ancient and conserved calcium regulatory system of mineralization.

## Introduction

The molluscs are the most diverse group of animals after the insects and the shell is their calcareous exoskeleton. This mineralized structure is mostly composed (>95%) of calcium carbonate crystals complexed within an organic protein matrix and emerged early in their evolution but was subsequently lost in some mollusc lineages^1,2^. The shell sustains the molluscs soft-body and is a protective shield against predators and environmental aggressions, but it is also a reservoir of minerals. Mollusca shell growth depends on the secretory activity of the mantle, a ciliated integument, and the mantle edge is the most active region in shell production^1–6^. The mechanisms underlying shell production in molluscs are of considerable interest, i) from a biomimicry perspective for biomineralization, ii) to identify novel calcium regulatory factors of biomedical interest and iii) to establish the likely impact of environmental change (ie. pH, salinity and temperature) on shell production and mollusc survival^7–10^.

Bivalves are sessile filter feeders and build shell using calcium (Ca^2+^) and bicarbonate (HCO_3_^−^) acquired from their environment or food. The Ca^2+^ and HCO_3_^−^ reaches the shell producing mantle via the haemolymph and also from the direct contact with seawater^1^. In the mantle, ion pumps, channels and enzymes regulate the secretion of Ca^2+^ and HCO_3_^−^ to the extrapallial space (a narrow isolated space between the shell and mantle) where shell fabrication occurs in the presence of matrix proteins^1,11–15^. Although proteins are a minor component of the shell they are responsible for the synthesis of CaCO_3_ crystals and depending on the shell matrix proteins, form either calcite (prismatic layer) and/or aragonite (nacreous or internal lustrous layer) mineralized structures^1,2^. The process of shell formation is tightly controlled by the mantle but the factors essential for this process remain largely unknown. Shell/mantle proteomes and mantle transcriptomes have yielded candidates for the proteinaceous shell scaffold but regulatory factors are less well defined and since conservation across bivalves is relatively poor there is still no consensus model for shell formation in bivalves^3,4,14,16–30^.

In terrestrial vertebrates’ bone mineralization and remodelling are well characterised processes and tightly linked to calcium homeostasis. In vertebrates, the bone serves as a vast reservoir of calcium and, in humans, the maintenance of normal calcium and phosphate levels in the body involves the coordinated action of calcitonin (CALC), a single-chain peptide that is secreted by the parafollicular cells of the thyroid and parathyroid hormone (PTH) that is produced by the parathyroid glands. CALC is a hypocalcaemic hormone and functions to reduce plasma calcium levels, while PTH is a hypercalcaemic hormone and increases plasma calcium concentrations^31–33^. They act on target tissues to regulate calcium uptake and reabsorption and control the function of bone forming osteoblasts and bone resorbing osteoclasts, respectively. Specific receptors for CALC (CALCR) and PTH (PTHR) have been described and their function characterized and they are members of a family of conserved seven transmembrane proteins, family B G-protein coupled receptors (GPCR) (a.k.a., Secretin-GPCRs)^34–36^. In humans, 15 peptide-binding family B GPCRs exist and based on sequence similarity and the activating ligands are classified into 5 main subfamilies. Two receptors of the CALCR subfamily (CALCR and CALCRLR) and of the PTHR subfamily (PTHR1 and PTHR2) have been characterized in humans. A higher level of signalling flexibility exists since it can be influenced by GPCR signalling via either heterotrimeric G proteins or by β-arrestins that modulate the potency and efficacy of the agonists^37^. Furthermore, interaction of GPCRs with other receptors or GPCR-Interacting Proteins (GIPs) can modulate receptor selectivity^38^. In the case of family B GPCRs they interact with the receptor activity modifying protein (RAMP) family. An example of the consequence of RAMP1 or RAMP2 binding to CALCR, is the shift from high affinity binding of CALC to binding of amylin^39^. For receptor activation, the N-terminal region of the peptide triggers intracellular signalling and two pathways are activated either, adenylate cyclase, which results in the increase of cAMP or phospholipase C, which causes mobilization of intracellular calcium (Ca^2+^)^40^.

Homologues of the vertebrate family B GPCRs have been described in nematodes and arthropods but remain unexplored in molluscs, the second most diverse and specious animal group^34,41–43^. In molluscs, putative CALCRs have been described in a few species and their expression in the mantle has made them candidate regulatory factors for shell mineralization. In the Pacific oyster (*Crassostrea gigas*) CALCR-like and CALC-like precursors are expressed in the mantle and they have been associated with ion balance^44,45^. In the mantle transcriptome of the Antarctic clam (*Laternulla eliptica*) putative CALCR-like and PTHR-like receptors were also reported^4^. Binding sites for CALC-like peptides were found in the gills and mantle of bivalves and cephalopods^46–49^ and peptides that are similar in structure to the human CALC were predicted *in silico*^42^ and identified by transcriptomics and proteomics in the Yesso scallop (*Patinopecten yessoensis*) nerve ganglia^50^. The presence of other members of the family B GPCRs in molluscs remains unknown as does the potential involvement of GIPs in modulating receptor selectivity.

In this study the aim was to improve understanding of mantle physiology and identify putative regulatory factors of shell production. The potential contribution of the family B GPCRs to shell biomineralization in molluscs was determined. Taking advantage of mollusc genomes and available mantle edge transcriptomes^3^ we identified mollusc family B GPCRs and ligands and provide a comparative analysis with vertebrates. We characterized the function of the CALC-system in the marine bivalve, the Mediterranean mussel (*Mytilus galloprovincialis)*, which by functional homology with the vertebrate CALC-system, is a strong candidate regulator of shell mineralization.

## Results

### Lophotrochozoan family B GPCRs

**Figure 1:**
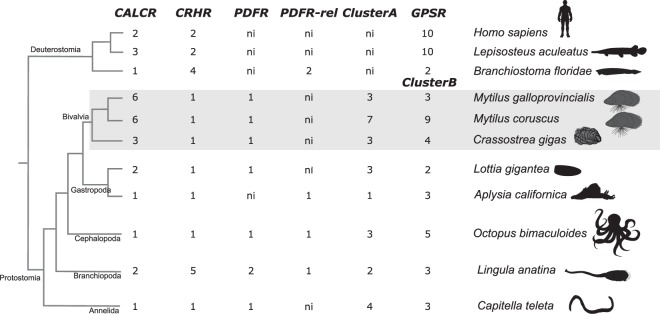
Number of Calcitonin receptors and other family B GPCRs in molluscs and other lophotrochozoans. Receptors were identified based on searches against available genomes and mussel (Mediterranean mussel, *Mytilus galloprovincialis* and hard-shelled mussel, *Mytilus coruscus*) mantle edge transcriptomes. Vertebrates (human, *Homo sapiens*, and spotted gar, *Lepisosteus aculeatus*) and invertebrate deuterostome (amphioxus, *Branchiostoma floridae*) family B GPCR members are represented for comparison. Family B GPCR subfamilies: Corticotropin-Releasing Hormone Receptor (CRHR), Pigment Dispersing Factor Receptor (PDFR), Pigment Dispersing Factor Receptor-related (PDFR-rel), Cluster A and Cluster B receptors. GPSR represents the deuterostome Glucagon (GCG), Parathyroid Hormone (PTH) and Secretin (SCT) receptor subfamilies that are homologues of the invertebrate Cluster B. A dendrogram depicting the current accepted evolutionary species relationship is represented. ni- not identified. Bivalves are highlighted in grey. Searches were performed in February/March 2019. Drawings were made using Inkscape, 2.7.11 (https://inkscape.org).

Family B GPCRs were identified in molluscs and other lophotrochozoans (Fig. 1, Supplementary Table 1). In the species analysed multiple members for CALCR, Cluster A and Cluster B subfamilies were found. In molluscs CALCR gene/transcript number per species is variable and the largest number of receptors were found in mussels where six were retrieved from the mantle edge transcriptomes (Fig. 1). A unique CRHR-like gene was found in molluscs, but five were retrieved from the lamp-shell brachiopod and PDFR/PDFR-related receptors were identified in only a few species (Fig. 1). In contrast, multiple members of Cluster A and Cluster B (homologues of vertebrate Glucagon/Parathyroid/Secretin receptor, GPSR) subfamilies exist, and Cluster B contained the greatest number of genes (Fig. 1, Supplementary Table 1). No receptors were retrieved from the blunt gaper (*Mya truncata*) mantle transcriptome. The predicted protein sequences of mollusc CALCRs and other family B GPCRs retrieved from mantle transcriptomes are available in Supplementary Data 1.

### Lophotrochozoan family B GPCR phylogeny

**Figure 2:**
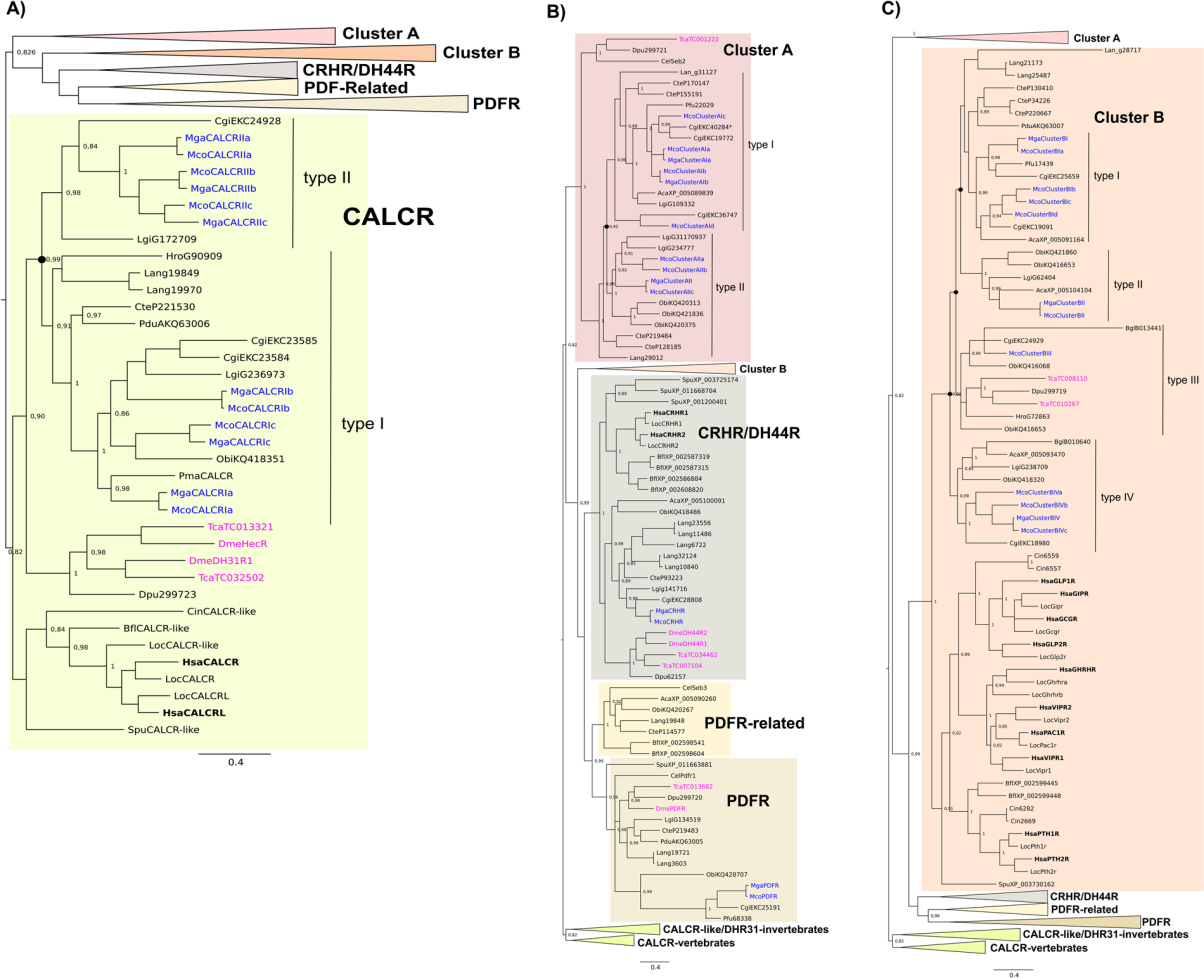
Phylogenetic trees of the lophotrochozoan CALCR and other family B GPCRs. The consensus tree was constructed with the BI method and was mid-rooted and the probability support values at nodes for the main clades are indicated. Three subsets of the same phylogenetic tree show the details of different family members and they are highlighted with different coloured boxes: (**A**) CALCR, (**B**) Cluster A, CRHR/DH44R, PDFR-related and PDFR and (**C**) Cluster B. The Pacific oyster CALCR-like sequence (Cgi, EKC40284) previously described^45^ is indicated by “*” and groups within Cluster A. To facilitate interpretation the positions of the Mediterranean mussel (*Mytilus galloprovincialis*, Mga) and hard-shelled mussel (*Mytilus coruscus*, Mco) receptors are highlighted in blue, the human (*Homo sapiens*, Hsa) in bold and the arthropod (fruit-fly, *Drosophila melanogaster*, Dme and flour beetle, *Tribolium castaneum*, Tca) in pink. Duplication events in molluscs that led to the two CALCR types and other family B GPCR types are indicated at the tree branches by a full circle. The ML trees are available as Supplementary Figure 1. The sequences included in the phylogenetic trees are listed in Supplementary Table 1.

Phylogenetic analysis of the lophotrochozoan family B GPCRs revealed that they shared a common ancestral origin with the receptor homologues found in vertebrates and in other protostomes (nematode and arthropods) (Fig. 2A–C). Both Bayesian Inference (BI) and Maximum Likelihood (ML) trees had similar branching topologies (Fig. 2, Supplementary Figure 1) and receptor clustering agreed with the previously proposed theories about family B GPCR evolution^43^. The distribution of the mollusc family B GPCRs confirmed that they possess members of the five vertebrate and invertebrate receptor subfamilies. In lophotrochozoans some gene families expanded and subsequently evolved via lineage and species-specific gene duplication events. Cluster A and Cluster B were the most diverse subfamilies as they contained the largest number of receptors identified. Within all the family B GPCR subfamilies the Mediterranean and hard-shelled mussel homologues clustered together due to their evolutionary proximity.

The mollusc CALCRs shared a common ancestral origin with the deuterostome CALCR and insect DH31R/HecR (Fig. 2A). Within the CALCR cluster the arrangement of the lophotrochozoan members suggested that two main receptor sub-clusters (two types) exist: CALCR-type I and CALCR-type II and that they have emerged from a specific gene duplication event early in this lineage (Fig. 2A). Distribution of the six Mediterranean and hard-shelled mussel and other mollusc CALCRs suggests that receptor gene expansion occurred within each species. The mussels possess the most diverse repertoire of CALCR and the characteristics of each was subsequently analysed. Based on phylogeny the receptors were named CALCRIa, CALCRIb and CALCRIc in the type I cluster and CALCRIIa, CALCRIIb and CALCRIIc within the type II cluster. Several members of the Cluster A subfamily were found in lophotrochozoans including the molluscs (Fig. 2B) and two main receptor sub-groups Cluster A type I and Cluster A type II exist. The Pacific oyster gene that was previously identified and assigned to CALCR^45^, grouped with members of the Cluster A type I subfamily in our analysis (Fig. 2B). Clustering of the mollusc CRHR-like confirmed that a single gene copy exists and that the 5 brachiopod receptors are the result of species-specific duplications (Fig. 2B). No bivalve homologues of the invertebrate PDFR-related receptors were found and only putative PDFR sequences were identified. In octopus, the brachiopod and annelid both PDFR and PDFR-related members exist (Fig. 2B). The majority of the mollusc sequences identified clustered within Cluster B, which is the most diverse group. The disposition of the sequences within the phylogenetic tree suggests that four main receptor sub-clusters (type I, II, III and IV) exist in molluscs and resulted from lineage and species-specific duplication events (Fig. 2C).

### Sequence conservation of mollusc CALCRs and CALC-like precursors

The bivalve CALCRs share 44–50% aa similarity with the human homologues and the most highly conserved regions were the 7 TM domains (Supplementary Table 2). Several residues in the large N-terminal extracellular domain that in mammals are linked to receptor function and structure, including the six conserved cysteines and the aspartate (D) residue before the motif C-W and the amino acid motifs C-W, C-P and G-x-W (where x represents any amino acid) were also conserved in molluscs^51,52^ (Supplementary Figure 3). The isoleucine (I) and alanine (A) residues located before the first cysteine that are essential for peptide binding and receptor expression at the cell surface were also conserved in some Mollusca sequences^53^ (Supplementary Figure 3).

**Figure 3:**
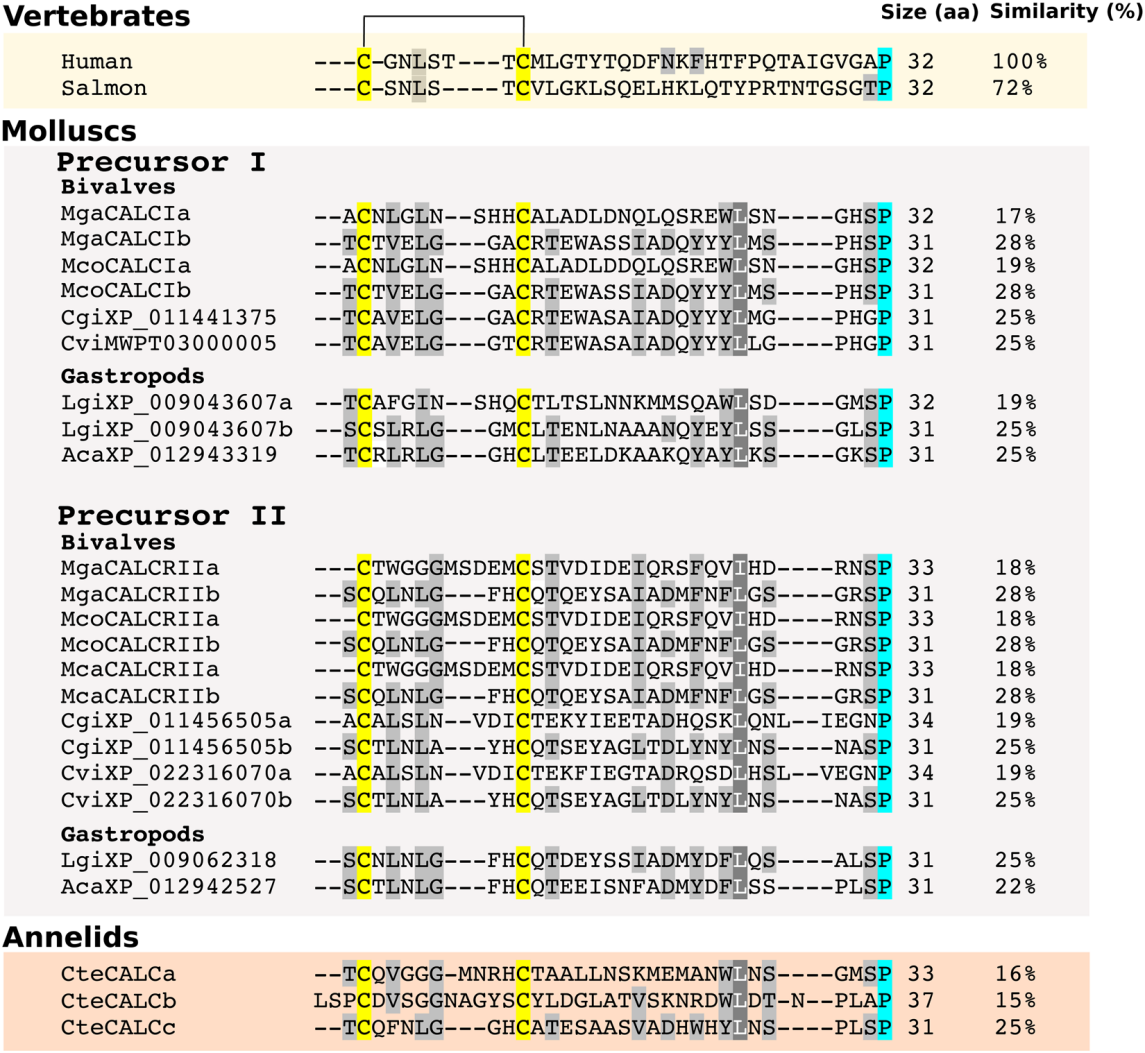
Multiple sequence comparison of the molluscan putative mature calcitonin-like peptide sequences with human and salmon calcitonins. The deduced calcitonin-like peptides from the Mediterranean mussel (Mga), hard-shelled mussel (Mco), Pacific oyster (Cgi) and eastern oyster (Cvi) as well as the gastropod owl limpet (Lgi) and California sea hare (Aca) are represented and are grouped as precursor I or precursor II types based on their sequence similarity (Supplementary Table 3). The three peptides deduced from the annelid (*Capitella teleta*, Cte) calcitonin-like precursor are also included for comparison. The conserved cysteine residues are annotated in yellow and the cysteine disulphide bridge is represented . The C-terminal conserved proline amide is annotated in blue. Amino acids shaded in grey indicate the conservation between the different sequences. The predicted size of the mature peptides (aa) and their percentage (%) aa sequence similarity with human calcitonin is indicated. The deduced peptide sequences were retrieved from the peptide precursor sequences available in Supplementary Figure 2.

In lophotrochozoan, transcripts and genes for putative CALC-like peptide precursors were found (Supplementary Figure 2). In the mussel mantle edge transcriptomes and in the Pacific oyster and eastern oyster two precursor transcripts were identified and they were designated *CALCI* and *CALCII*. Searches in the Pacific oyster genome revealed that they correspond to different genes: *CALCI* in scaffold43406 and *CALCII* in scaffold954. In the gastropods, the owl limpet and the sea hare snail, two precursors also exist. Based on the consensus proteolytic cleavage sites and peptide sequences, 4 putative CALC-like peptides may be produced *in vivo* from the Mediterranean and hard-shelled mussel CALC-like precursors: two peptides (CALCIa and CALCIb) are encoded by the *CALCI* precursor and two peptides (CALCIIa and CALCIIb) by the *CALCII* precursor (Supplementary Figure 2, Fig. 3). However, no consensus proteolytic cleavage site was identified at the N-terminus of CALCIIa but all the conserved motifs of the “typical” CALC peptide were present. The predicted mussel mature peptides are 31 to 33 aa in length and are of a similar size to the salmon and human peptides (32 aa) (Fig. 3). The mussel deduced mature peptides share low similarity (17–28% aa) with human CALC but contain the conserved functional residues such as, the two cysteines in the N-terminus and an amidated proline at the C-terminus (Fig. 3). The Mediterranean and hard-shelled mussel homologue peptides are identical except for CALCIa that differs at only one aa position (Fig. 3, Supplementary Table 3). In other molluscs the organization of the two CALC-like precursors differ from the mussel and in the Pacific oyster, eastern oyster and owl limpet they have distinct coding potential: one codes for two peptides and the other a single peptide. In the annelid *C. teleta* a unique peptide precursor gene that codes for three different CALC-like peptides was identified (Fig. 3, Supplementary Figure 2).

### Gene neighbourhood analysis

The gene environment of mollusc *CALCR-like* (type I and II) and the *CALC-like* precursor (type I and II) were similar to the vertebrate (human and spotted gar) and fruit-fly genomes (Supplementary Figures 4 and 5). At least seventeen *CALCR* flanking gene families, that descended from common ancestry were shared between molluscs and vertebrates (Supplementary Figure 4). Comparison of the mollusc gene environment with the fruit-fly *DH31R* gene on chromosome 2 L revealed three shared genes that also have homologues (*trim71* and *cnga*) in the genome of the spotted gar but not in human (Supplementary Figure 4). Mapping of the Pacific oyster and owl limpet suggests that the two mollusc *CALCR-like* gene types likely arose from a gene duplication event prior to the bivalve and gastropod divergence. At least five genes were shared between human and mollusc homologue genome regions for the* CALC* peptide gene (Supplementary Figure 5). No homologous genes with the fruit-fly genome region that contains the *DH31* peptide precursor gene (2 L) were identified.

### Peptide precursor and receptor expression in mussel

**Figure 4:**
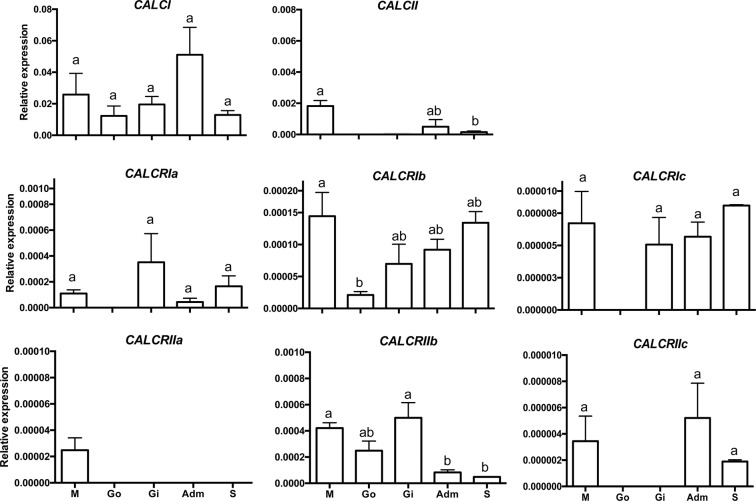
Tissue distribution of the Mediterranean mussel (*Mytilus galloprovincialis*) calcitonin peptide precursors and receptors. Gene expression levels were determined by quantitative PCR and normalized using the geometric mean of two reference genes (*ef1α* and *18 s*). The results are represented as the mean ± SEM of three (n = 3) biological replicates. One-way Anova and Tukey’s multiple comparison test was used to assess differences in transcript expression with Prism GraphPad v5 software. Bars with different letters are significantly different (p < 0.05). M: mantle (posterior edge); Go: Gonads; Gi: Gills; Adm: Adductor muscle ; S: Stomach.

**Figure 5:**
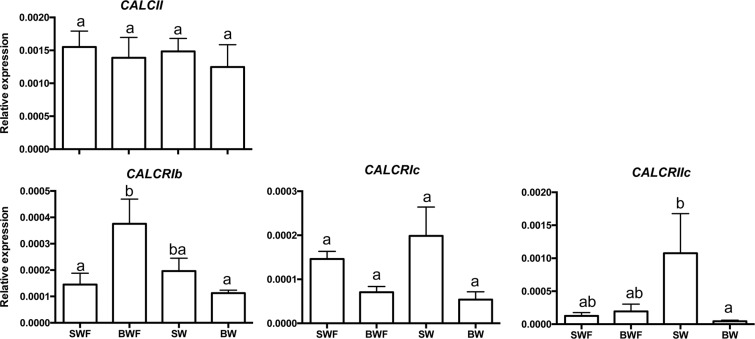
Effect of salinity and food on the expression of calcitonin-like precursors and receptors in the mantle edge of the Mediterranean mussel. Changes in expression levels were investigated in the posterior mantle edge exposed to full (SW) or decreased (brackish water, BW) water salinity in fed or fasted (F) mussels. Preliminary q-PCR analysis suggested that no significant differences between samples were likely to exist for *CALCI* and *CALCRIa*, *CALCRIIa* and *CALCRIIb* and so they were not further analysed. Gene expression levels were normalized using the geometric mean of two reference genes (*ef1α* and *18 s*). The results are represented as the mean ± SEM (n = 5–6 biological replicates). One-way Anova and Tukey’s multiple comparison test was used to assess differences in transcript expression with Prism GraphPad v5 software. Bars with different letters are significantly different (p < 0.05).

The widespread tissue distribution of the six *CALCR* and two *CALC-like* precursor transcripts in the Mediterranean mussel suggests that they may have a number of different physiological functions; the gene transcripts for the peptide precursors were more abundantly expressed than the receptors (Fig. 4). In the hard-shelled mussel transcriptome CALC-like precursors were also more abundant (approximately 10-fold) than receptors (Supplementary Figure 6). In the Mediterranean mussel both peptide precursor and receptors were amplified from the mantle (Fig. 4). *CALCI* was the most expressed peptide precursor and was found in all tissues analysed while *CALCII* was amplified in the mantle and adductor mussel and was of very low abundance or not amplified in the remaining tissues (Fig. 4). Of the six mussel receptors, *CALCRIb* and *CALCRIIb* had the most widespread distribution and were amplified in all the tissues analysed. *CALCRIa* and *CALCRIc* levels were undetectable in gonads, *CALCRIIc* was not amplified in the gonads and gills and *CALCRIIa* was only amplified in the mantle. Salinity and feeding affected mussel receptor gene expression. *CALCRIb* expression was significantly increased (p < 0.05) in BWF suggesting that receptor expression is regulated both by water salinity and feeding. Expression of *CALCRIIc* was significantly down-regulated (p < 0.05) in BW compared to SW. Expression of *CALCII* and *CALCRIc* were not significantly modified by salinity or feeding (Fig. 5).

### Mussel CALCR-like receptors functional characterisation

**Figure 6:**
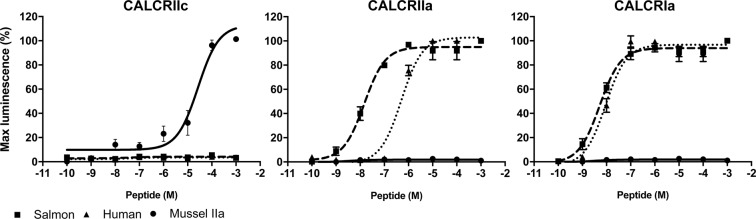
Stimulation of the mussel CALCRs with mussel, human and salmon calcitonin peptides. Dose-response profiles of the three peptides that activated the mussel CALCR-like receptors. Receptors were stimulated with 1 μM to 0.01 nM of the mussel calcitonin-like peptide CALCIIa (•) or salmon (□) and human (Δ) calcitonin peptides and the mobilization of intracellular calcium was measured using HEK 293 cells co-transfected with the apoaequorin vector. The mussel CALCRIIc was exclusively activated by the mussel CALCIIa. The peptide potency profile for the mussel CALCRIIa was salmon » human (p < 0.001) and for CALCRIa was salmon > human (p < 0.05) and these receptors were not activated by the mussel peptide. Data was calculated as a percentage (%) of the highest response obtained (100% activation). Values represent the mean ± SEM of at least three independent experiments performed in duplicate and EC_50_ values were calculated from dose–response curves obtained from the three independent assays. Differences between EC_50_ were analysed using a two-tailed unpaired t-test.

Activation of the mussel CALCR-like expressed in mammalian HEK 293 cells was characterised (Fig. 6, Supplementary Figure 7). All mussel receptors were stimulated by human and salmon CALC via the cAMP pathway, but not by the mussel peptides (Supplementary Figure 7 A). Mussel CALCR-like responded to vertebrate or mussel CALC-like peptides with increased intracellular calcium release (Supplementary Figure 7B). Dose-response curves were further characterised for the latter intracellular signalling pathway, for the mussel CALC peptide-receptor pair and for two other receptors for comparison with the human and salmon peptides (Fig. 6). The other mussel receptors chosen were CALCRIa and CALCRIIa and they were selected based on i) their high responsiveness to the vertebrate peptide in the calcium mobilization assay (preliminary results), ii) their phylogenetic position, as they represent members of the two different CALCR-types (Fig. 2) and iii) their expression in the mantle [CALCRIa is the type I receptor most expressed in the mantle and CALCRIIa was only amplified in the mantle (mantle-specific)] (Fig. 4). Our dose-response analysis confirmed that both human and salmon CALC peptides activated the mussel CALCRIa and CALCRIIa but not CALCRIIc that was only activated by mussel CALCIIa (EC_50_ = 2.6 × 10^−5^ M) (Fig. 6). In addition, the dose-response curves of CALCRIa and CALCRIIa receptors differed between the vertebrate peptides. The CALCRIa receptor was more strongly (p < 0.05) activated by the salmon calcitonin (EC_50_ = 5.0 ×10^−9^ M) than the human calcitonin (EC_50_ = 9.5 ×10^−9^ M). Similarly, the CALCRIIa receptor response to salmon calcitonin (EC_50_ = 1.4 ×10^−8^ M) was significantly stronger (p < 0.001) than to human calcitonin (EC_50_ = 5.0 × 10^−7^ M).

### Effect of CALCIIa peptide on calcium transport in the mussel mantle edge

**Figure 7:**
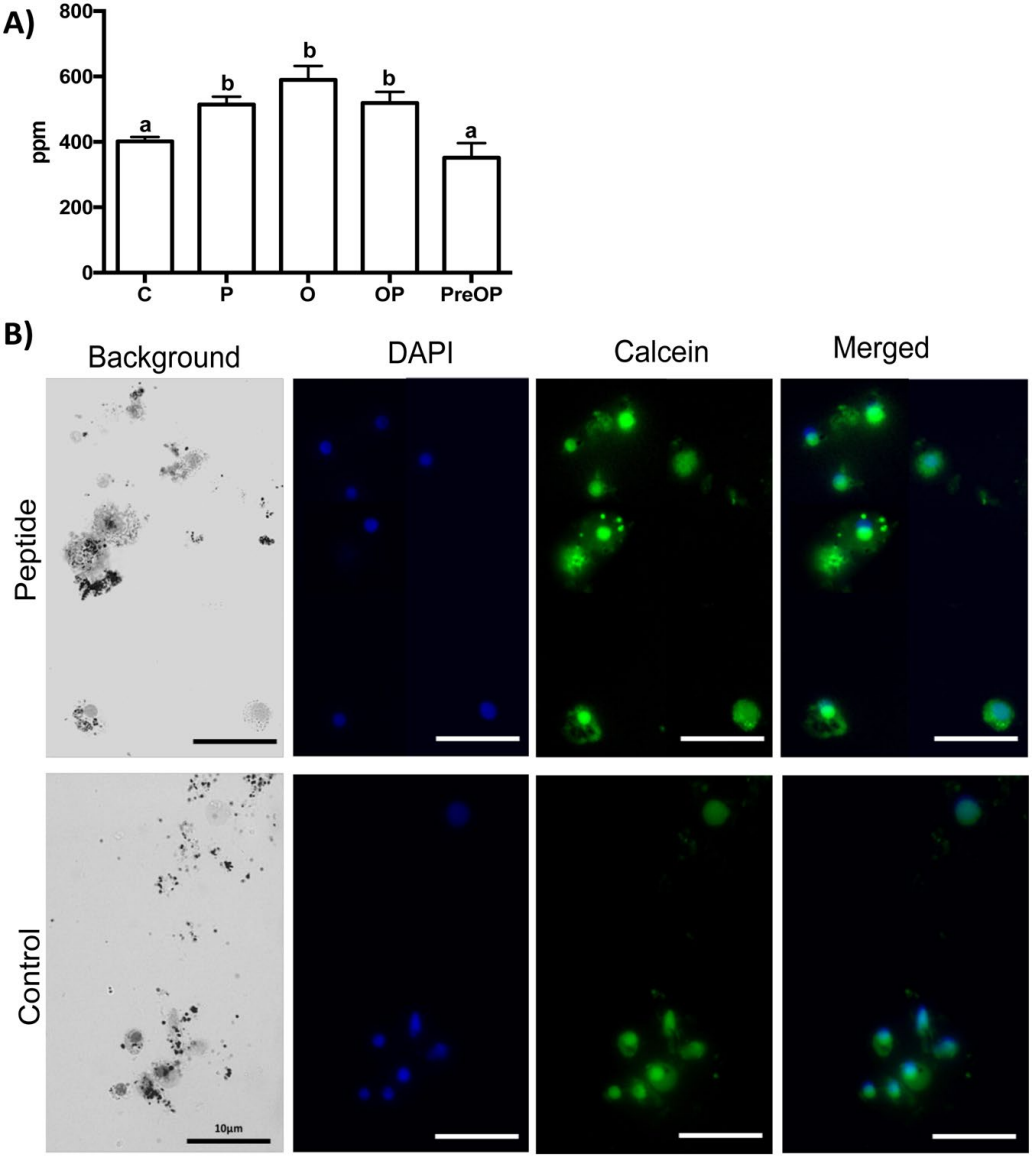
Effect of the mussel calcitonin-like peptide on (**A**) calcium (ppm) concentration in the mantle edge and (**B**) calcium uptake by mantle cells of the Mediterranean mussel. (**A**) The quantity of calcium (ppm) present in the mantle edge after mussel CALCIIa peptide incubation was measured using a Microwave Plasma-Atomic Emission Spectrometer (MP-AES, Agilent) at 393.366 nm. A standard curve for calcium was performed before analysing the samples. *In vitro* mantle assays with CALCIIa peptide and with ouabain and control (culture medium and mantle) were repeated on at least 3 independent occasions. For each assay at least ten replicates were performed and the mantle edge distal to the umbo was used (one mantle fragment per replica). Prism GraphPad v5 software was used to assess the significance of differences between the groups using a One-way Anova and Tukey’s multiple comparison test. Bars with different letters are significantly different (p < 0.05). C - control (no peptide or ouabain); P - peptide (10 *μ*M); O - ouabain (2 *m*M), OP - ouabain (2 *m*M) and peptide (10 *μ*M); PreOP *-* pre -stimulation for 1 hour with the peptide followed by 4 hours incubation with OP. (**B**) Digital photographs of the mussel mantle cells in the presence (CALCIIa, 10 *μ*M) or absence (control) of the mussel CALCIIa peptide. Calcium ions were stained with 50 mM of Calcein (green) and cell nuclei (blue) with DAPI (Acros Organics). An increase in cellular fluorescence represents an increase in calcium uptake. Fluorescence of the CALCIIa peptide stimulated cells was 1.9 times higher than the control. The average mantle cell size was 3–4 μm and fluorescent images were taken using a Leica DM IL microscope coupled to a Visicam HDMI 6 digital camera and photographs were analysed using ImageJ software (v. 1.52a) to calculate the fluorescence intensity and for image overlay. The scale bar is indicated.

Incubations of fragments of the *M. galloprovincialis* mussel mantle edge with CALCIIa significantly (p < 0.05) stimulated the cellular uptake of calcium in relation to the control (Fig. 7A). After 4 h incubation with CALCIIa (P), the mantle of the peptide stimulated group contained 513.38 ± 24.33 ppm calcium when compared to 400.67 ±13.83 ppm in the control (C). Ouabain (O) also significantly (p < 0.05) increased mantle calcium uptake compared to the control (588.71 ± 42.92 ppm) and a similar effect was observed when CALCIIa and ouabain were added simultaneously (OP, 518.40 ± 34.16 ppm) (Fig. 7A). However, when the mantle was first primed with CALCIIa and subsequently incubated with CALCIIa and ouabain the uptake of calcium was similar to the control (PreOP, 351.07 ± 44.56 ppm). Incubation of the mantle fragments with Verapamil (data not shown) did not change the calcium concentration relative to the control. In isolated mantle cells a 1.9 times increase in the intensity of fluorescent calcium staining was observed in cells that were exposed to CALCIIa compared to control mantle cells (mean of the corrected tested cell fluorescence (CTCF) for control = 1405154 and peptide challenged = 2654581) Fig. 7B.

### Enzyme activity in mantle

**Figure 8:**
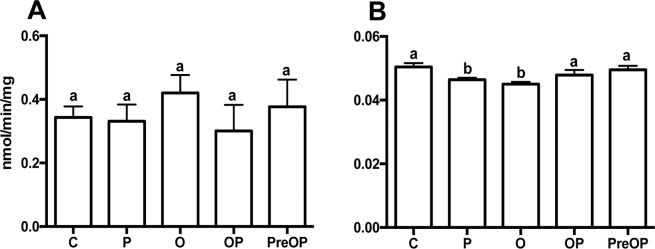
Activity of enzymes involved in biomineralization in the mantle after incubation with the mussel peptide. (**A**) Esterase activity and (**B**) Acid phosphatase activity. Enzyme activity was quantified in protein extracts from the mantle of control or challenged samples that were incubated with the mussel CALCIIa peptide. Values represent the mean ± SEM (CA, n = 6 and TRAP, n = 10) performed in duplicate. Prism GraphPad v5 software was used to assess the significance of the differences between the groups using One-way Anova and Tukey’s multiple comparison test. Bars with different letters are significantly different (p < 0.05). C - control; P - peptide (10 *μ*M); O - ouabain (2 *m*M), OP - ouabain (2 *m*M) and peptide (10 *μ*M); PreOP *-* pre stimulation for 1 hour with the peptide and 4 hours incubation with OP.

The activity of the alpha-carbonic anhydrase (CA) enzyme that is involved in the production of bicarbonate ions^54–56^ and acid phosphatase (AP) that is associated with regulation of shell mineralization in the mollusc mantle^57^ was measured in the mussel mantle edge after exposure to CALCIIa (Fig. 8A,B, respectively). Transcripts for both enzymes were found in the mussel mantle edge transcriptomes^58^ (and data not shown). No change in CA esterase activity was detected (Fig. 8A) but AP activity was significantly decreased (p < 0.05) by CALCIIa and ouabain exposure compared to the control (Fig. 8B). The results indicate that the CALCIIa stimulated increase in mantle calcium was inversely proportional to AP enzyme activity (Fig. 8B).

## Discussion

Homologues of vertebrate calcium regulatory factors exist in molluscs but their involvement in mantle function and shell building is unknown. In this study to better understand mantle physiology and shell biomineralization we have characterized the invertebrate CALC system and identified related members of the family B GPCRs in the mussel mantle.

In molluscs a similar system to the vertebrate CALCR and CALC peptides was found and the phylogeny and gene linkage indicate they shared a common ancestry with the vertebrate homologue system but evolved under different pressure. Two types of receptors that arose from a gene duplication event persisted in mollusc genomes, but the evolution of the receptor precursor molecules was lineage-and species specific. In arthropods, DH31R is the sequence homologue of the vertebrate CALCRs and in nematodes no putative *CALCR* genes were described^42^. The two type of *CALCR* genes found in molluscs, annelids and in the branchiopod suggests that the ancestral lophotrochozoan *CALCR* genes duplicated earlier and subsequently expanded within each species. In the present study, expression and receptor activation studies suggested that the two ancestral receptor types evolved differently and have probably acquired distinct functions in the mussel mantle. The sequence similarity between the identified mussel and oyster receptors and the functional studies performed by others suggests that the oyster *CALCR* genes probably evolved through a similar process^44^. Analysis of the neighbouring genes of the bivalve and gastropod *CALCR* revealed that after the first gene duplication the receptor genome regions evolved differently and under distinct pressures as only a single gene was conserved between these molluscs. Nonetheless, the organization of the *CALCR* gene environment in molluscs is more closely related to the vertebrate homologue genome regions than to the insect *DH31R* loci. In insects DH31R is associated with water balance regulation and Na^+^ transport and not with tissue mineralization^59^, and recently it has also been associated with regulation of insect body thermal rhythms^60^.

Characterization of the lophotrochozoan family B GPCRs confirmed that members descended from a unique bilaterian ancestral gene by gene duplication prior to the protostome-deuterostome split^43^. In common with nematodes and arthropods, the clustering of the mollusc and other lophotrochozoan receptors suggests that they probably arose by lineage and species-specific gene duplications driven in part by organismal adaptation to the environment. Members of the six main family B GPCR receptor subfamilies , including homologues of the recently identified nematode and arthropod Cluster A, Cluster B and PDFR-related groups, also exist in molluscs, annelids and in brachiopods. In molluscs a large expansion of Cluster A and Cluster B members occurred. In insects and nematodes, where the members of these receptor groups were initially described ligands have yet to be identified and they remain orphans and their function is unknown^43^. Identification in the bivalve mantle of Cluster A and Cluster B receptor transcripts provides a candidate tissue for future studies to explore their function. In our study no homologues of insect DH31R were identified and they seem to be arthropod-specific. Recently in the Pacific oyster seven CALCRs were described and in phylogenetic analysis some grouped with insect DH31R (Cragi-CTR3 to 6)^44^. However, in our study they clustered with the invertebrate Cluster A and are not authentic homologues of vertebrate CALCR. One of the oyster DH31R-like transcripts was found to be responsive to water salinity^45^ and its role in ion balance opens-up new avenues of investigation for Cluster A and B receptor function.

In mammals, CALC is a small 32 amino acid peptide secreted by the thyroid C-cells and has a C-terminal amidated proline and an N-terminal circular ring structure formed by a disulphide cysteine bridge, which is essential for bioactivity. The CALC peptide is a member of a family that share low sequence conservation but have high structural conservation and bind to common receptors. Peptide affinity for the receptors in mammals is modulated by a group of transmembrane proteins, the Receptor Activity-Modifying Proteins (RAMPs)^61^. Mammalian CALC is produced by alternative splicing of the calcitonin gene which also encodes for the structurally related, calcitonin gene-related peptide (CGRP), a potent vasodilator released by the central and peripheral nervous system^62^. In mammals, a second CALC gene (*CALC II*) exists but only encodes CGRP^63^. In molluscs, no RAMPs were identified (results not shown) and the two CALC-like precursors share a similar organization to the human CALC precursor^44^. The low overall amino acid sequence similarity but the conservation in the mollusc peptides of key amino acids responsible for vertebrate CALC bioactivity suggests that receptor activation functions have been maintained and may explain why the vertebrate peptides were able to activate all the mussel receptors. Good conservation of the Mollusca receptors and the vertebrate homologues suggests that during their evolution they were under conservative selective pressure presumably due to their essential physiological role. Only one of the identified mussel peptides, CALCIIa, activated one of the mussel receptors (CALCRIIc) and the cognate peptides for the remaining receptors remain to be identified but may be the other mussel CALC peptides that were not tested in our study. Additional CALC-like precursors may also be identified when the hard-shelled mussel genome and improved genome assembly for the Mediterranean mussel are available. Both human and mollusc CALC peptides activated the Mollusca CALCRs and this activation did not require the co-expression of RAMPs either in mussel (our study) or in oyster^44^.

In this study we demonstrated, for the first time that, in common with vertebrates, the CALC-system in bivalves is coupled to the regulation of calcium transport. A constant supply of calcium and bicarbonate ions is needed for marine bivalves to build their shell. Both ions are either obtained from the seawater or from food and uptake occurs across through the gills and gut and also the mantle. In the latter case ions are transferred via the haemolymph into the extrapallial space that is in contact with the epithelial cells on the dorsal side of the mantle^1^. Bicarbonate is also produced by reverse hydration of metabolic carbon dioxide (CO_2_) via the action of the mantle carbonic anhydrases. The transfer of calcium across the outer mantle epithelium (OME) to the extrapallial space is suggested to be finely regulated by ion transporters and calcium channels together with passive paracellular ion transfer^12^. In the mantle transcriptomes of *C. gigas* and *P. fucata* plasma membrane Ca^2+^ ATPase (PMCA), a sodium-calcium exchanger (NCX) and several calcium channels similar to the human homologues were detected^12,23,64–66^ and in *M. edulis* an increase in gene expression of sarco/endoplasmic reticulum Ca^2+^-ATPase (SERCA) and NCX was associated with periods of active larval calcification^67^. Recently we demonstrated in *C. gigas* that voltage gated calcium channels (VGCCs), such as PMCA and NCX participate in Ca^2+^ transfer across the OME^11^. The observed increase in calcium levels in the mussel mantle edge caused by the sodium-potassium ATPase ion pump inhibitor, ouabain, is presumably due to inhibition of NCX^68,69^. The results from the present study corroborate the results from electrophysiology and suggest an important role for NCX in shell formation through regulation of calcium movements in the Mediterranean mussel mantle. The mussel CALCIIa peptide stimulated uptake of calcium by the mantle. This confirms that the mechanism of mantle calcium regulation and transfer to the extrapallial space in bivalves and potentially in other molluscs is a complex process and that GPCRs may regulate calcium movements across the mantle. When the mantle was exposed simultaneously to mussel CALCIIa and ouabain, intake of calcium by the mantle was similar to what occurred when the peptide and ouabain were assayed individually. In contrast, ion extrusion occurred when the mantle was previously primed by the peptide. This suggests that in the presence of an inhibitor of the sodium-potassium ion pump the mussel peptide inhibits calcium efflux from the mantle to achieve the same calcium concentrations as the control. The exact mechanism by which mussel CALC peptides regulate calcium movements across the mantle requires much more investigations as does the coupling with ion transporters and shell matrix protein secretion.

Mussel calcitonin peptide also seems to regulate mantle enzyme activity and decreased mantle acid phosphatases activity (AP) *in vitro*. APs are an ancient group of metalloenzymes that are poorly characterized in bivalves. Because of their insensitivity to tartrate inhibition, they are also known as tartrate-resistant acid phosphatases^70,71^. AP in vertebrates is associated with bone resorption and is a marker of increased osteoclast activity^72^. The few studies of AP in molluscs suggest that its activity is associated with shell remodelling and resorption and in gastropod snails AP is present in shell forming tissue^73^ and in the pearl oyster is expressed in the mantle and inhibits calcium carbonate crystal formation *in vitro*^57^. In bivalves a function in immune defence has also been postulated^74^. A role for AP in mussels has not been described but the inhibition of enzyme activity when calcium increases in the mantle suggests it may have a similar role to that found in other molluscs and vertebrates in preparing the matrix for crystal nucleation and growth. In contrast, neither of the bivalve CALCs or ouabain modified the activity of carbonic anhydrases, essential enzymes for shell production, suggesting that in contrast to human erythrocytes, bicarbonate production is not regulated by calcium transport^75^. The activity of alkaline phosphatase (ALP), another marker of mineralization in vertebrates^76–78^, was not determined as only negligible enzyme activity was detected in the mantle edge (data not shown). If peptide function is conserved across other regions of the mussel mantle remains to be explored.

## Conclusion

In molluscs the mantle plays a major role in shell formation but the mechanism by which shell is produced remains poorly understood. Here we demonstrate that in molluscs and other lophotrochozoans a system similar to the vertebrate CALC system exists and is expressed in the mussel mantle edge and likely shares conserved functions. A functional peptide-receptor pair (CALCRIIc and CALCIIa) was found and the mussel peptide significantly stimulated calcium uptake and interfered with the activity of enzymes involved in biomineralization in the mantle. We hypothesize that in mussel and potentially in other bivalves the CALC-like system may act as a local regulator of calcium ion transport in the mantle. Taking into consideration the ubiquitous presence in the genome/transcriptome of bivalves and other molluscs of the CALC-like system and its localization in the mantle we propose that it has a key role in regulating shell biomineralization. A different number of CALCR and mature CALC-like peptides evolved across the molluscs. The retention of the CALC-system and the lineage and species-specific evolution in molluscs, accompanied and possibly contributed to the diverse and exuberant shell structures and shapes acquired since they emerged during the Cambrian epoch^30,79^. The other Mollusca family B GPCRs in the mussel mantle are targets for future studies of mantle physiology.

## Materials and Methods

### Database mining

#### Receptors

To explore the mollusc family B GPCR system and its potential involvement in mantle function, mantle transcriptomes available from the Mediterranean mussel (*Mytilus galloprovincialis*, SRP 063654^3^, the hard-shelled mussel (*Mytilus coruscus*, kindly provided by Yifeng Li and Jin-Long Yang, SHOU, China), the Antarctic clam (*Laternula elliptica*)^4^, scallop (*Pecten maximus*^80^) and the blunt gaper (*Mya truncata*^81^) were explored. Transcripts were identified by searching with the human CALCR (ENSG00000004948), calcitonin-gene related peptide receptor (CALCRL, ENSG00000064989), corticotropin hormone receptor 1 (CRH1R, NP_004373.2), pituitary adenylate-cyclase activating polypeptide receptor (ADCYAP1R1, NP_001109.2) and the parathyroid hormone receptor 1 (PTH1R, NP_000307.1) and the sequences from the insect, flour beetle (*Tribolium castaneum*) Diuretic hormone 31 (DH31R, TC002694), Pigment Dispersing factor receptor (PDFR, TC013682), Diuretic hormone 44 (DH44R, TC007104), HecR (TC013321), Cluster A (TC001222) and Cluster B (TC010267)^43^ using local BLAST searches. Sequence hits with a cut off <e^−30^ were retrieved and their identity confirmed by comparison against human (taxid:9606).

The bivalves, Pacific oyster (*Crassostrea gigas)* and Japanese pearl oyster (*Pinctada fucata*), the gastropods, California sea hare (*Aplysia californica*), owl limpet (*Lottia gigantea*) and fresh water snail (*Biomphalaria glabrata*) and the cephalopod (*Octopus bimaculoides*) genomes were explored using a similar strategy to that outlined above. The available Mediterranean mussel (ASM167691v1^82^) genome was also interrogated but the sequence hits obtained were very short and this made gene prediction difficult and the data was not used. To better understand receptor gene evolution in the molluscs, searches were extended to the genomes of evolutionary related species such as the brachiopod lamp shell (*Lingula anatina*) and two annelids, the polychaete worm (*Capitella teleta*) and leech (*Helobdella robusta*). Data available at NCBI for the marine worm (*Platynereis dumerilii*,) and basal deuterostomes: the echinoderm, purple sea urchin (*Strongylocentrotus purpuratus*); the cephalochordate, amphioxus (*Branchiostoma floridae*) and the tunicate (*Ciona intestinalis*) were also searched. Receptors from the genome of the ray-finned fish, the spotted gar (*Lepisosteus oculatus*) were also retrieved for comparative purposes. All searches were performed in February/March 2019.

### Peptide precursors

Homologues of the vertebrate mature peptide that activate family B GPCRs were procured in molluscs. The deduced mature CALC-like peptide sequences of the owl limpet (XP_009062318, XP_009043607) and polychaete worm (*Capitella teleta*)^42^ were used to search the bivalve mantle transcriptomes and genomes of mollusc and other lophotrochozoan as described above. Data available at the NCBI for molluscs (taxid:6447) was also interrogated. The identity of the retrieved peptide precursors was inferred by blast searches against the human genome and the localization of the mature peptide within the complete peptide precursor was predicted by: a) the identification of monobasic, dibasic or tribasic peptide proteolytic consensus cleavage sites (RR, KR, KK); b) the presence of two N-terminal conserved cysteines and c) the existence of a C-terminal amide glycine (Supplementary Data 1).

### Sequence alignments and phylogenetic analysis

The sequence of mussel transcripts were used to predict the proteins (https://web.expasy.org/translate/) and the initiation start and stop codons were chosen, respectively as the first methionine and first stop codon that provided the correct reading frame. When no initial methionine was found (incomplete sequences) the deduced sequences were retrieved from the frame that produced the largest protein. Amino acid sequence similarities/identities were calculated using GENEDOC software (http://www.nrbsc.org/gfx/genedoc). Sequences were aligned using the MUSCLE algorithm^83^ available from the ALIVIEW platform 1.18^84^. Phylogenetic analysis of the mollusc CALCR-like receptors included the homologues from vertebrates and other invertebrates but also other mollusc family B GPCRs and their homologues. Receptor sequences from the fruit-fly (*Drosophila melanogaster*), flour beetle (*Tribolium castaneum*), nematode (*Caenorhabditis elegans*) and water flea (*Daphnia pulex*) were also included (Supplementary Table 1).

The receptor sequence alignment was manually inspected to eliminate incomplete and/or highly divergent sequences and gaps. As a result, several of the identified mollusc sequences were not considered in the analysis (Supplementary Table 1). The final edited alignment (184 sequences and 324 aa positions) was used for phylogenetic analysis with the Maximum Likelihood (ML) and Bayesian Inference (BI) methods. ML tree were performed with the PhyML 3.0 algorithm^85^ with the SMS automatic model selection according to the AIC (Akaike Information Criterion)^86^. The chosen model was a WAG substitution model^87^ and reliability of internal branching was accessed using 100 bootstrap replicates. The BI tree was performed in MRBAYES 3.2^88^ using a WAG substitution model with 1,000,000 generations. ML and BI trees were mid-rooted, and both were displayed with FIGTREE 1.4.3 (http://tree.bio.ed.ac.uk/software/figtree).

### Gene linkage

The gene environment of the Pacific oyster and owl limpet *CALCR-like* genes were compared with the homologue genome regions in human and spotted gar. The deduced protein sequences of the genes that map close to the oyster and owl limpet receptors were retrieved and use as bait to identify homologues in the vertebrate genomes (www.ensembl.org). The assembled genome of the fruit-fly (*D. melanogaster*) was also searched using a similar strategy.

### Animal manipulation and sampling

Mediterranean mussels (*M. galloprovincialis*) were obtained from a local producer in the Ria Formosa (Olhão, Portugal). Mussels (length 3.76 ± 0.27 cm, wet weight 6.39 ± 1.27 g) were transported live to the Centre of Marine Sciences (CCMAR) where they were cleaned and acclimatized for a week in aerated tanks with filtered natural sea water (SW) prior to experimentation. Before tissue dissection animals were anaesthetized by immersion in a magnesium chloride (28 mg/L in SW) solution for 30 min. Gonads, gills, adductor muscle, stomach and the mantle edge from the region most distant from the umbo (referred here as the posterior region) of the left valve were dissected out and snap frozen in liquid nitrogen for RNA extraction.

### Salinity challenge

To investigate the role of the mussel CALC-like system in ion balance, ion availability was modified either by changing water salinity of the bathing water or by removing food and changes in mantle transcripts of the peptide precursor and receptor were quantified. A salinity stress was established by maintaining the mussels in brackish water (BW) for two-weeks. BW (salinity 18 ppt) was obtained by diluting (1:1 v/v) filtered SW (salinity, 37 ppt) with deionised water (0.054 µS, ELIX, MILLIPORE, USA). The SW used was collected from the Ria Formosa and filtered using a WHATMAN 0.45 µm filter. A feeding challenge was also applied and animals were divided into four groups: SWF (SW with food), BWF (BW with food), SW (SW without food) and BW (BW without food). All the experiments were performed using mussels that were collected at the same time. For each experimental condition mussels (n = 12 animals each) were divided between three tanks with 4 animals in each. Experiments were performed in 2 L plastic tanks with 1 L of SW or BW constantly aerated at 20 ± 1 °C and under a 12 light: 12 dark photoperiod (October 2016). Half the tank water (0.5 L) was renewed every 2 days and the pH was monitored (8.1 ± 0.1). Mussels in the SWF and BWF were fed daily with a fresh microalgae mix (*Nannochloropsis sp., Tetraselmis sp*. and *Isochrysis sp.-* 4,6 ×10^5^ cells/ml). No mortality was observed during the experiment and no changes in shell length or animal weight were observed.

### RNA extraction and cDNA synthesis

Total RNA from adult mussel tissues (gonads, gills, stomach, adductor muscle and the posterior mantle edge, n = 3) was extracted from 40 mg of tissue using the Extrazol reagent (DNA GDANSK, Poland). Tissues were homogenized manually using a plastic pestle and purified using a Maxwell RNA purification kit (PROMEGA, Spain) and treated with 1 U DNase (DNA-free Kit, AMBION, UK) for 30 min at 37 °C in accordance with the manufacturer’s instructions. Mantle total RNA from ion challenged animals was extracted with an E.Z.N.A kit (VWR, USA) and DNase I treatment was performed directly on the columns. For cDNA synthesis, DNase treated total RNA (500 ng) was denatured at 65 °C for 5 min and quenched 5 min on ice. Reactions were carried out for a 20 μl final volume with 10 ng of pd(N)6 random hexamers (Jena Bioscience, Germany), 2 mM dNTPs (THERMOSCIENTIFIC, USA), 100 U of RevertAid Reverse Transcriptase and 8 U Ribolock RNAse inhibitor (THERMOSCIENTIFIC). Reaction conditions were: 10 min, 20 °C; 60 min, 42 °C and 5 min at 70 °C. The integrity of the synthetized cDNA was assessed by amplification of the mussel ribosomal subunit 18 s rRNA (Table 1) using the following thermocycle: 95 °C, 3 min; 25 cycles (95 °C, 20 sec; 60 °C, 20 sec; 72 °C, 20 sec); 72 °C, 5 min.

**Table 1:** List of the primer sequences.

Name	Sequence (5′-3′)	Temp (ºC)	Efficiency (%)	R^2^
**Relative expression**				
Receptors				
CALCRIa-Fwd	TGTCGGATTTGTCAGACAATCC	62	98.4	0.99
CALCRIa-Rev	TTTTGTGTTAAAGCCTGGGAG			
CALCRIb-Fwd	GACACCGGAAGAAACGTTATC	56	106.4	0.99
CALCRIb-Rev	AGCTATTGATTGCCTAGGATCC			
CALCRIc-Fwd	TGCAATGCTACATCAGACACA	60	93.5	0.99
CALCRIc-Rev	CTGGCAATGAGTATAATCTCCTC			
CALCRIIa-Fwd	GCTTGGAGTTCATAGACAGCG	62	98.7	0.99
CALCRIIa-Rev	TTCAATAAATTGGTCTGGAGG			
CALCRIIb-Fwd	ACAATGGACAGTTTCCATAGG	62	92.7	0.98
CALCRIIb-Rev	TGGTAGCTAGAAATATTTCGGT			
CALCRIIc-Fwd	CACAATGGAACTATTAGTGCAC	60	99.8	0.99
CALCRIIc-Rev	GATAGTCCCAGCAATCATACC			
Calcitonin precursors				
CALCI-Fwd	TGGTTGAAACTTACATGTGGTT	60	95.5	0.99
CALCI- Rev	CATTCTTCTTCAATGACGTCA			
CALCII- Fwd	AAACGGGCGTGCAATCTTG	62	96.7	1.0
CALCII -Rev	CGAATGTTCTTTAGGTCAAGGC			
Housekeeping genes				
18S-Fwd	GTGCTAGGGATTGGGGCTTG	58	99.2	0.99
18S-Rev	TAGTAACGACGGGCGGTGTG			
Ef1alpha-Fwd	GAAGGCTGAGCGTGAACGTG	58	104.2	0.99
Ef1alpha-Rev	TCCTGGGGCATCAATAATGG			
**Full-length receptor isolation**				
CALCRIa -Fwd	ATGTCGGATTTGTCAGACAATCCT			
CALCRIa -Rev	TCACGTGACCGATGTAGCAACACC			
**Receptor cloning**			**Restriction sites**	
CALCRIa-Fwd	GCGGGATCCATGTCGGATTTGTCAGAC		BamHI	
CALCRIa-Rev	GCGCTCGAGTCACGTGACCGATGTAG		XhoI	
CALCRIb-Fwd	CGCGGATCCATGACCCCAGAGGAAA		BamHI	
CALCRIb-Rev	GCGTCTAGATCAGACATCGCACACG		XbaI	
CALCRIIa-Fwd	GCGAAGCTTATGGAGAACGGCACCTC		HindIII	
CALCRIIa-Rev	GCGGCGGCCGCTCACACGTTAGACTCGA		NotI	
CALCRIIb-Fwd	CGCGGATCCATGGCCGCTGACCGAA		HindIII	
CALCRIIb-Rev	GCGTCTAGATCAGGATTTGATCTGA		XbaI	
CALCRIIc-Fwd	CGCGGATCCATGGCATACAACGGCA		HindIII	
CALCRIIc-Rev	CGCTCTAGATCACACGTCAGTTGTC		XbaI	

The annealing temperature and the efficiency (%) of the primer pairs and the linearity R^2^ of the standard curve are indicated.

### Quantitative expression

Changes in gene expression were assessed by quantitative real-time PCR (q-PCR) using SsoFast EvaGreen Supermix (BIO-RAD, Portugal). A 10 µl final reaction volume was prepared with 200 nM of forward and reverse gene specific primers (Table 1) and 2 µl of template cDNA (diluted 1:5). The mussel elongation factor 1-alpha (*ef1α*) and 18 S ribosomal subunit (*18 s*) transcripts were used as housekeeping reference genes (cDNA was diluted 1:100 and 1:1000, respectively) as their expression levels did not vary between samples. Duplicate reactions were performed (<5% variation between replicates) using a CFX Connect Real-Time PCR Detection System for 96-well microplates (BIO-RAD). Cycling conditions were 95 °C, 30 sec; 44 cycles (95 °C for 5 sec and 10 sec at the appropriate primer annealing temperature, Table 1). To detect non-specific products and primer dimers melting curves were performed. q-PCR efficiencies and R^2^ (coefficient of determination) were established (Table 1) and expression levels were calculated based on the relative standard curve method and data was normalized using the geometric mean of both reference genes. All amplicons were sequenced to confirm reaction specificity.

**Table 2:** Mussel calcitonin-like peptides.

*CALCIa*	H-ACNLGLNSHHCALADLDNQLQSREWLSNGHSP-NH_2_
*CALCIIa*	H-CTWGGGMSDEMCSTVDIDEIQRSFQVIHDRNSP-NH_2_
*Human*	H-CGNLSTCMLGTYTQDFNKFHTFPQTAIGVGAP-NH_2_
*Salmon*	H-CSNLSTCVLGKLSQELHKLQTYPRTNTGSGTP-NH_2_

Mussel peptides were synthesised using as the amino acid sequence the deduced mature peptide determined by analysis of the full-length peptide precursor transcripts (Supplementary Figure 2). The complete sequence of the human and salmon peptides is also indicated

### Receptor isolation and cloning

The complete sequence of the Mediterranean mussel *CALCRIa* was amplified using specific primers (Table 1) from cDNA of a tissue mixture (mantle, adductor muscle, gills, gonads) using a proofreading DNA polymerase (iProof BIORAD, Portugal) and cloned into the pGEMT-easy vector (PROMEGA, Spain). The Mediterranean mussel *CALCRIIa* transcript that was found in the mantle transcriptome was incomplete and lacked the N-terminus (by comparisons with the hard-shelled mussel homologue) and so the remaining sequence (from 1–393 bp) was deduced from the hard-shelled mussel homologue receptor and the receptor was synthesised and supplied by GENESCRIPT (USA). The complete transcripts for mussel *CALCRIc*, *CALCRIIb* and *CALCRIIc* were only found in the hard-shelled mussel mantle transcriptome and homologues were only found in the Mediterranean mussel genome as they were absent from the species mantle transcriptome and so they were also synthesised and supplied by GENESCRIPT (USA) using the hard-shelled mussel receptor sequences as template. The mussel *CALCRIb* transcript was incomplete and lacked both N and C-terminal regions and it was not possible to obtain the complete transcript either by searching both mussels mantle transcriptomes or the Mediterranean mussel genome and so it was not produced. All synthetized receptors were provided in the pUC57 cloning vector by the company and receptor codon usage was optimized to favour a high level of expression in mammalian cells.

Receptors were amplified from the cloning vector using a proofreading DNA polymerase (iProof, BIORAD) with specific primers and a restriction enzyme digestion site was incorporated into the 5’ region (Table 1) to allow directional cloning of the receptor into pcDNA3.1/V5-His TOPO expression vector (INVITROGEN, USA). The successfully amplified products were gel extracted (GE, UK), digested with the appropriate restriction enzyme (fast-enzymes, THERMO SCIENTIFIC) at 37 °C for 30 min, column purified and ligated into the purified and digested pcDNA3.1/V5-His TOPO expression vector. Ligation reactions were performed using T4 DNA ligase (THERMOSCIENTIFIC) at 4 °C overnight and the reaction mix was used to transform competent DH5-alpha cells. The recombinant plasmids were isolated using the standard alkaline lysis method and sequenced to confirm their appropriate integration in the vector. Mussel receptors were not cloned in frame with any reporter proteins.

### Mammalian cell cultures and transfections

Mammalian cell cultures and transfections followed the methodology described by^89,90^. Mammalian HEK293 cells (ECACC, UK) were used and they were maintained in complete Dulbecco’s modified Eagle’s medium (DMEM, 4.5 g/l glucose, 110 mg/l sodium pyruvate and L-glutamine, SIGMA-ALDRICH, Spain) supplemented with 10% sterile foetal bovine serum (FBS), 0.1% penicillin: streptomycin antibiotic mix (10,000 U:10 mg/ml, Sigma) and 250 μg /ml sterile filtered 1:100 amphotericin B solution (SIGMA-ALDRICH) in a humid 5% CO_2_ incubator (HERAEUS, Portugal) at 37 °C. On the day prior to transfection, 2–3 × 10^5^ HEK293 cells were seeded on 6 well plates (SARSTEDT, Portugal) and transfected with Fugene HD transfection reagent (2 DNA: 4 Fugene ratio, PROMEGA) following the manufacturer’s protocol. Seventy-two hours after transfection, 800 μg/ml of the antibiotic Geneticin (SIGMA-ALDRICH) was added to the medium to select and isolate stable receptor cell lines. Cell recovery was monitored daily and the medium was changed every two days until no cell death was observed. Establishment of stable cell lines was confirmed by PCR using specific primers for each receptor.

### CALC peptides

The Mediterranean mussel CALCIa and CALCIIa mature peptides were synthesized (purity > 95%) and modifications included the disulphide bond, N-terminal acetylation and C-terminal amidation (GL BIOCHEM, China) (Table 2). The CALCIa peptide (size 32 aa) corresponded to the mature peptide sequence. For the deduced CALCIIa peptide no consensus dibasic proteolytic site was predicted at the N-terminus and so the peptide synthesized (size 33 aa) was from the first Cysteine of the disulphide bond to the C-terminal amidated proline. Synthesis of the remaining two mussel peptides was unsuccessful. The human and salmon CALC peptides were purchased from BACHEM (Germany).

### Receptor pharmacological characterization

HEK293 cells stably expressing five mussel receptors (*CALCRIa*, *CALCRIc*, *CALCRIIa*, *CALCRIIb* and C*ALCRIIc*) were stimulated with the two mussel peptides and the vertebrate (human and salmon) calcitonins. Receptor activation was assessed *in vitro* by measuring two intracellular signalling pathways: a) production of cAMP and b) mobilization of calcium (Ca^2+^). The cellular response was initially assessed using 10 *μ*M of each peptide. HEK293 cells stably transfected with the empty pcDNA3.1 vector was used as the negative control in both assays.

### cAMP assay

The amount of cAMP produced was determined using a competitive immunoassay with a cryptate labelled anti-cAMP antibody (CISBIO, France) and followed the methodology describe by^89,90^. Briefly, approximately 15,000 stably transfected mammalian HEK293 cells were assayed per well and cell-peptide incubations were performed for a final reaction volume of 20 *μ*l in white 384 well small Volume HiBase Polystyrene microplates (GREINER, Germany). Prior to the peptide assay, cells were resuspended in 1 x PBS with 1 *m*M of 3-isobutyl-1- methylxantine (IBMX, SIGMA-ALDRICH) and incubated for 5 min at 37 °C. Calcitonin peptides were diluted in 1 x PBS/ 1 *m*M IBMX and added to the cells for 30 min at 37 °C in a CO_2_ incubator. Receptor stimulation was analysed in a Biotek Synergy 4 (BIOTEK, USA) plate-reader using the 620/10 nm and 665/8 nm filters. Three independent experiments were performed for each receptor using triplicate reactions for each assay.

### Calcium mobilization assay

Stable cell lines expressing the mussel receptors were transiently co-transfected with the mitochondrial targeted apo-aequorin protein cloned in pcDNA3.1. Two days after transfection cells were washed, counted and resuspended in 1 ml of DMEM /F12 Ham (without phenol red)/0.1% BSA supplemented with 2 µM coelenterazine (SIGMA-ALDRICH). Cell suspensions were incubated for 3 hours at RT in the dark with gentle agitation. To obtain a final concentration of 5 ×10^5^ cells/ml they were subsequently diluted in DMEM / F12 Ham / 0.1% BSA and incubated for an extra hour at RT in the dark with gentle agitation. The injector system of the Biotek Synergy 4 microplate reader (USA) was used to inject 50 µl of the cell suspensions into wells containing 50 *μ*l of the diluted peptide in DMEM / F12 Ham / 0.1% BSA. Bioluminescence assays were measured using white, 96 well plates with a flat bottom (INVITROCELL, Portugal) and luminescence was recorded every 2 s for 30 s. Peptide induced Ca^2+^ responses were normalized to the total Ca^2+^ response after addition of Triton X-100 (0.1%). Peptide potency was determined as the total area under the activation curve. Data was calculated as a % of the highest response (100% activation). Assays were performed in duplicate and EC_50_ calculated from dose–response curves obtained in three independent assays (GRAPHPAD PRISM, 7.0a).

### *Ex-vivo* peptide incubations

#### Mantle tissue

The posterior mantle edge region of the Mediterranean mussel was collected and washed in culture medium. The composition of the culture medium was as described in^91^ (30% L15 medium (Sigma) and 70% SW) but the sea water used was collected from the mussels natural environment, filtered (0.22 µm) and the pH was adjusted to 8.0–8.1 with 5 M NaOH (average water pH for the Ria Formosa, Portugal). The culture medium was supplemented with heat inactivated 2% FBS (SIGMA-ALDRICH), 0.1% penicillin: streptomycin antibiotic mix (10.000 U:10 mg/ml, SIGMA-ALDRICH) and 250 μg/ml sterile filtered 1:100 amphotericin B solution (Sigma-Aldrich). The posterior mantle edge from mussels was collected into culture medium and cut into fragments of approximately 1–2 mm in length. For each assay, sixty mussels were used. Mantle fragments were randomly assign to different wells of a 96 well-plate (Sarstedt, Portugal). The experimental groups included, a) the control group (C) that contained the mantle tissue and medium and b) the stimulated group where the mantle was maintained in medium supplemented with 10 *μ*M of mussel CALCIIa peptide (the concentration was chosen based on the EC_50_ value of receptor activation studies). Experiments with both groups were carried out simultaneously, with the same reagents and under identical conditions except for the presence of the peptide in the stimulated group. Incubations were carried out for 4 hours at 19–20 °C. Mantle incubations were also performed (under identical conditions to the control and CALCIIa experiments) with pharmacological inhibitors that interfere with calcium transport^12^: Verapamil (200 µM, V, SIGMA-ALDRICH), a voltage-gated calcium channel blocker and ouabain (2 mM, O, Sigma-Aldrich), a Na/K-ATPase (NKA) inhibitor that reduces the activity of sodium-calcium exchanger (NCX). The concentrations of the inhibitors were based on^12^. In another assay tissue fragments were primed with the CALCIIa peptide (10 *μ*M) for 1 hour and subsequently incubated for a further 4 hours with medium supplemented with the CALCIIa and Verapamil or ouabain. The incubation period was determined based on the results of preliminary assays (data not shown).

### Mantle cells

Mantle cells were isolated from Mediterranean mussel mantle edge explants of the posterior region and were incubated with the CALCIIa peptide. A mantle cell control group was performed simultaneously and under the same condition but the only difference what that no peptide was added. Mussel mantle cells were obtained under aseptic conditions by mincing the posterior edge of the mantle into 1-mm pieces in culture medium and transferring them into 6-well cell culture plates at 19–20 °C to allow cell release. After 4 days, cells that migrated out of the tissue were collected and incubated in the presence (stimulated group) or absence (control group) of CALCIIa (10 *μ*M) for 4 hours. To detect calcium ions, cells were at the same time incubated with 50 mM of calcein (SIGMA-ALDRICH) a fluorescent dye used for the determination of calcium prepared in sterile filtered SW. Cells were subsequently washed with 1 x PBS and the nuclei stained with 4′,6-Diamidine-2′-phenylindole dihydrochloride (DAPI) (300 nM, ACROS ORGANICS) in PBS for 5 minutes at room temperature in the dark. Digital images were captured at 63x total magnification with a LEICA DM IL microscope coupled to a VISICAM HDMI 6 camera. Photographs were analysed for image overlay and to calculate fluorescence intensity (corrected total cell fluorescence, CTCF) using ImageJ software (v. 1.52a). The CTCF was measured using the following formula: CTCF = Integrated density- (Cell area x mean fluorescence of background) for the control and peptide stimulated groups.

### Calcium quantification in the mantle tissue

The amount of calcium (ppm) in the mantle and culture medium was quantified using a Microwave Plasma-Atomic Emission Spectrometer (MP-AES, AGILENT). After incubation with CALCIIa peptide the mussel posterior mantle edge was washed in 1x PBS, dried at 60 °C for at least for 2 days and ground into a fine powder using a mortar and pestle. The tissue powder was weighed and digested at RT for 2 days in concentrated HNO_3_ (0.1 g of tissue/ml). The culture medium was also collected and centrifuged for 15 min, 12,000 rpm at RT to separate cells and tissue fragments and 20 µl of the clean supernatant was digested with 80 µl of concentrated HNO_3_. The reaction was performed for 2 days using the same conditions as for tissue digestion. All samples were subsequently kept at 4 °C and assayed within 4 days of hydrolysis. For MP-AES analysis tissues and medium were diluted 1:50 and 1:500, respectively with Normatom water (VWR, Portugal)/5% HNO_3_ to a final volume of 2 ml and calcium ion content determined at 393.366 *n*m. A standard curve for calcium was performed and used to establish calcium concentration.

### Mantle enzyme activity

#### Esterase activity

Esterase activity in the mussel posterior mantle edge (n = 6/experimental group) was quantified using a colorimetric assay that measures the conversion of the substrate 4-Nitrophenyl acetate to p-nitrophenolate^92^. Assays were performed using the method described in^58^. The amount of p-nitrophenolate produced was quantified using a standard curve constructed using p-nitrophenol (from 0 to 200 µM). Bovine CA isoenzyme II (0.1 mg/ml) (SIGMA-ALDRICH) was used as a positive control.

### Acid phosphatase activity

Acid phosphatase activity of the mantle protein extracts (n = 6/ group) was determined using 96 well-plates (GREINER, Germany) and a tartrate resistant acid phosphatase (TRAP) assay as described in^93^. Briefly, 10 *μ*l of the mantle protein extract was added to 190 *μ*l of TRAP buffer (20 *m*M paranitrophenyl (pNPP, SIGMA-ALDRICH), 20 *m*M tartrate in 0.1 M Na-acetate buffer, pH 5.3) and incubated for 20 min at RT with agitation. Reactions were stopped with 2 M NaOH and measured at 405 *n*m using a microplate reader (BIOTEK SYNERGY 4). The amount of pNPP converted into p-nitrophenol (pNP) was calculated using a pNP standard curve.

### Statistical analysis

Results are presented as the mean ± SEM. Statistical differences were detected using One-Way ANOVA followed by Tukey’s multiple comparison test. For EC_50_ comparisons a two-tailed unpaired t-test was performed. The significance cut-off was taken at p < 0.05. The data analysis was executed using GRAPHPAD PRISM version 7.0a for Mac OS X (USA, www.graphpad.com).

## Supplementary information

Supplementary information.
