# Supplementary material for: The calcitonin-like system is an ancient regulatory system of biomineralization
Source: Sci Rep. 2020 May 5;10:7581. doi: 10.1038/s41598-020-64118-w (PMC7200681; doi:10.1038/s41598-020-64118-w)
Supplement: Supplementary file 1 — Supplementary information. [file 41598_2020_64118_MOESM1_ESM.docx]

**Supplementary Information**

**The calcitonin-like system is an ancient regulatory system of biomineralization**

João CR Cardoso*^1^, Rute C Félix^1^, Vinícius Ferreira^1^, MaoXiao Peng^1^, Xushuai Zhang^1^ and Deborah M Power*^1, 2, 3^

^1^Comparative Endocrinology and Integrative Biology, Centre of Marine Sciences, Universidade do Algarve, Campus de Gambelas, 8005-139 Faro, Portugal

^2^International Research Center for Marine Biosciences, Ministry of Science and Technology, Shanghai Ocean University, Shanghai, China

^3^Key Laboratory of Exploration and Utilization of Aquatic Genetic Resources, Ministry of Education, Shanghai Ocean University, Shanghai, China

* Corresponding authors

João CR Cardoso: jccardo@ualg.pt

Deborah M Power: dpower@ualg.pt

**Supplementary Tables - 3**

**Supplementary Figures - 7**

**Supplementary Data - 1**

**Supplementary Tables**

**Supplementary Table 1: Nomenclature and accession numbers (genes and transcripts) of the sequences retrieved for the phylogenetic analysis.** Sequences annotated in italics were not used for the phylogenetic analysis as they were incomplete. Transcript data was obtained from the mantle transcriptomes of the species indicated. The predicted protein sequences for family B GPCRs retrieved from the Mollusca mantle transcriptomes are available in Supplementary Data 1. Database searches were performed in February/March 2019. * deduced from the genome.

**Supplementary Table 2: Percentage of amino acid sequence identity/ similarity of the full-length calcitonin receptors found in mussel mantle transcriptomes with human calcitonin receptors.**

**Supplementary Table 3: Percentage of amino acid sequence identity between the Mediterranean mussel mature calcitonin-like peptides and those from other molluscs and an annelid.** The identity of peptides in other species was assigned based on the highest sequence identity with the Mediterranean mussel mature peptides and they are highlighted in bold.

**Supplementary Figures**

**Supplementary Figure 1: Phylogenetic trees of the Molluscan CALCRs and other family B GPCRs.** Trees were constructed using the Maximum likelihood method, a WAG matrix and 100 bootstrap replicates. The bootstrap support values at nodes for the main vertebrate clades are indicated. Three subsets of the same phylogenetic tree highlighted with different coloured boxes show in detail the different family members A) CALCR, B) Cluster A, CRHR/DH44R, PDFR-related and PDFR and c) Cluster B. To facilitate interpretation the positions of the Mediterranean mussel (*Mytilus galloprovincialis*, Mga) and hard-shelled mussel (*Mytilus coruscus*, Mco) receptors are highlighted in blue, the human (*Homo sapiens*, Hsa) in bold and the arthropod (fruit-fly, *Drosophila melanogaster*, Dme and flour beetle, *Tribolium castaneum*, Tca) in pink. Duplication events in molluscs that led to the two CALCR types and other family B GPCR types are annotated at the tree branch by a full circle. Accession numbers for the sequences used are provided in Supplementary Table 1. The Pacific oyster CALCR-like sequence (Cgi.EKC40284) previously described ^45^ is indicated with an “*” and is grouped within Cluster A in the phylogenetic tree.

**Supplementary Figure 2: Deduced mature protein sequences of the calcitonin-like precursors in molluscs and in an annelid.** The predicted mature calcitonin-like peptides are highlighted in bold and underlined, conserved cysteines (C) are in yellow, protease cleavage sites are shaded in red, the C-terminal proline (P) in green and the amidated glycine (G) is marked in blue. Accession numbers are indicated within brackets.

**Supplementary Figure 3:** **Sequence alignment of the mussel CALCR-like with the human CALCR and CALCRL**. The oyster Cgi.EKC23584 and Cgi.EK23585 CALCR-like were also included as they cluster in close proximity with the mussel receptors. The sequences of the hard-shelled mussel CALCRIb and of the Pacific oyster EKC24928 were not included because they are incomplete. Only the Mediterranean mussel CALCRIa is included because it was the only full-length receptor sequence retrieved from the mantle edge transcriptome of this species. Receptor sequences were aligned based on the phylogenetic tree grouping into type I and type II. The predicted localization of the TM domains are boxed in blue and are named. The conserved N-terminal cysteines are boxed in yellow and the consensus N-glycosylation sites (N-x-T/S) are boxed in red. The amino acid residues previously reported to play an important role in human CALCR and CALCRL function are annotated with “*”. Amino acids linked to function such as aspartate (D) before the C-W motif and the amino acid motifs C-W, C-P and G-x-W (where x represents any amino acid) that are crucial for ligand-binding in mammals were conserved in molluscs. The isoleucine (I) and alanine (A) residues located prior to the first cysteine that are essential for peptide binding and receptor expression at the cell surface were also conserved in some sequences. The predicted signal peptide sequence for the human receptors is underlined. Black and grey shading denote the level of amino acid conservation across the sequences.

**Supplementary Figure 4:** **Conserved gene synteny of the Pacific oyster and owl limpet containing CALCR-like type I and II genome regions with the vertebrate CALCR and fruit-fly DH31R chromosomes.** Many of the genes that are linked to CALCR in vertebrate genomes were present in the flanking region of CALCR-like in one or other of the molluscs analysed. Most of the shared genes were single copy (eg: *Mab21l2*, *chrn* and *TRIM*) with the exception of the specific protein (SP) transcript factor family members and Tubulin alpha-1A chain (*TUBA*), which expanded in vertebrate genomes probably as a consequence of the early vertebrate genome tetraploidization events. The CALCR flanking regions were poorly conserved between Pacific oyster and owl limpet and only the *MAP3K19* gene was shared and a homologue was also found in the spotted gar and human genomes. Horizontal lines indicate chromosome fragments and coloured arrows identify genes and their predicted orientation in the genome. The mollusc CALCR-like genes (type I or type II) are represented by red coloured arrows. Open red lined arrows represent other family B GPCR genes. Orthologue genes are represented by the same colour and their positions in the genome are indicated below (Mb). Only shared genes are represented. Mitogen-Activated Protein Kinase 19 (*MAP3K19*), Mab-21 Like 2 (*Mab21l2*), Specificity protein transcription factors (*SP*), Cholinergic receptors nicotinic subunits (*chrn*), Tailless (*tll*), Tripartite motif family (*TRIM*), Leucine Carboxyl Methyltransferase 2 (*lcmt2*), alpha-tubulin (*TUBA*), the solute carrier 6 (SLC6), solute carrier family 24 member 2 (*slc24a2*), Cytochrome b reductase 1 (*CYBRD1*), carboxypeptidase vitellogenic like (*CPVL*), Coiled-coil domain-containing protein 148 (CCDC148), Peptidyl-prolyl cis-trans isomerase (*CYPB1*), Nucleolar MIF4G domain-containing protein 1 (*NOM1*), Ankyrin repeat and IBR domain-containing protein 1 (*ANKIB1*), Cyclic nucleotide-gated cation channel subunit A (*cnga*). Drawings were made using Inkscape, 2.7.11 (https://inkscape.org).

**Supplementary Figure 5:** **Conserved gene synteny of the Pacific oyster and owl limpet containing CALC-like peptide precursor genome regions with the human and spotted gar.** At least five genes (*PIK3C2A*; *TRAF6*; *FAR1*; *CPSF7*; *SMTNL1*) were shared between human and molluscs. No homologue genes were found between the Pacific oyster and owl limpet CALC-like genome regions. The locus of the vertebrate and molluscan CALC peptide precursors is indicated by a red arrow with the exception of the Pacific oyster that is represented by a dashed arrow as no gene is predicted in the species genome. Horizontal lines indicate chromosome fragments and coloured arrows identify genes and their orientation in the genome. Orthologue genes are indicated by the same colour and their positions indicated below (Mb). Only shared genes are presented. Phosphatidylinositol-4-phosphate 3-kinase C2 domain-containing alpha polypeptide (*PIK3C2A*), TNF receptor associated factor 6 (*TRAF6*), Fatty acyl CoA reductase 1 (*FAR1*), Cleavage and polyadenylation specificity factor subunit 7 (*CPSF7*), Smoothelin-like protein 1 (*SMTNL1*), ribosomal protein S13 (*rps13*). Drawings were made using Inkscape, 2.7.11 (https://inkscape.org).

**Supplementary Figure 6: Relative abundance (fragments per kilobase of transcript per million, fpkm) of CALC-like and CALCR transcripts in the hard-shelled mussel (*Mytilus coruscus*) mantle edge transcriptome.** Abundance of CALC-like precursors and CALCR-like transcripts in the three different mantle edge regions (posterior, middle and anterior) is indicated (n = 4 cDNA libraries per region). C) schematic representation of a mussel to highlight the different mantle edge regions for which data was available.

Both CALC-like (A) and CALCR (B) were expressed in the posterior (blue), middle (orange) and anterior (grey) mantle edge regions. One-way Anova and Tukey’s multiple comparison test was used to assess differences in transcript expression. Bars with different letters are significantly different (p < 0.05).

**Supplementary Figure 7: Capacity of the mussel calcitonin-like and salmon and human calcitonin peptides to stimulate the mussel CALCR-like.** Mussel receptors were stably transfected in mammalian HEK 293 cells and subsequently stimulated with 10 *μ*M of the Mediterranean mussel or vertebrate (human and salmon) peptides. Receptor stimulation was measured by quantifying the production of the intracellular signalling molecules cAMP (A) and calcium (B). For calcium quantification the stable CALCR-like cells were co-transfecting with the apo-aequorin protein. All mussel receptors were stimulated in the presence of the vertebrate peptides. cAMP results represent the mean ± SEM of a single experiment performed in triplicate and data was calculated as a percentage (%) of the highest response obtained (100% activation) with Forskolin (10uM). Calcium mobilization results represent the outcome of a single experiment performed with six replicates and data was calculated as a percentage (%) of the highest response obtained (100% activation) with carbachol (100uM). Two mussel peptides were tested but only CALCIIa elicited a high response and so it was chosen for further analysis.

**Supplementary Data 1: Deduced protein sequences of the predicted family B GPCRs retrieved from the Mollusca mantle transcriptomes.** The sequences from the Mediterranean mussel (*Mytilus* *galloprovincialis*, Mga), hard-shelled mussel (*Mytilus coruscus*, Mco), Antarctic clam (*Laternula elliptica*, Lel) and scallop (*Pecten maximus*, Pma) are listed. * predicted from the Mga genome.

**Supplementary Table 1**

|  | **NOMENCLATURE** | **GENE** | **TRANSCRIPT** |
| --- | --- | --- | --- |
| **PROTOSTOMES** | | | |
| **Molluscs** | | | |
| **Bivalves** | | | |
| *Mytilus* *galloprovincialis (Mga)* | MgaCALCRIa  MgaCALCRIb  MgaCALCRIc  MgaCALCRIIa  MgaCALCRIIb  MgaCALCRIIc  MgaPDFR  MgaCRHR MgaClusterAIa  MgaClusterAIb  MgaClusterAII  MgaClusterBI  MgaClusterBII  MgaClusterBIII |  | Mga.17942  Mga.c90872*  Mga.c81686*  Mga.21503  Mga.c111933*  Mga.c97725*  Mga.14401  Mga.20948  Mga.22587  Mga.19893  Mga.27870  Mga.21652  Mga.8983  Mga.10532 |
| *Mytilus coruscus (Mco)* | McoCALCRIa  McoCALCRIb  McoCALCRIc  McoCALCRIIa  McoCALCRIIb  McoCALCRIIc  McoPDFR  McoCRHR McoClusterAIa  McoClusterAIb  McoClusterAIc  McoClusterAId  McoClusterAIIa  McoClusterAIIb  McoClusterAIIc  McoClusterBIa  McoClusterBIb  McoClusterBIc  McoClusterBId  McoClusterBII  McoClusterBIII  McoClusterBIVa  McoClusterBIVb  McoClusterBIVc |  | Mco.c106592  Mco.c90872  Mco.c81686  Mco.c112289  Mco.c111933  Mco.c97725  Mco.c165064  Mco.c105921  Mco.c95780  Mco.c93774  Mco.c102018  Mco.c58999  Mco.c81322  Mco.c88917  Mco.c122895  Mco.c109368  Mco.c6956  Mco.c8481  Mco.c62952  Mco.c94088  Mco.c132667  Mco.c82762  Mco.c107732  Mco.c88161 |
| *Crassostrea gigas (Cgi),* www.ensemblgenomes.org | CgiEKC25191  CgiEKC24929  CgiEKC25659  CgiEKC19091  CgiEKC36747  CgiEKC40284  CgiEKC19772  CgiEKC24928  CgiEKC23584  CgiEKC28808 | EKC25191.1  EKC24929.1  EKC25659.1  EKC19091.1  EKC36747.1  EKC40284.1  EKC19772.1  EKC24928.1  EKC23584.1  EKC28808.1  *EKC18980.1*  *EKC35246.1* |  |
| *Pinctada fucata (Pfu)*  http://marinegenomics.oist.jp/pearl/ version: 1.0 | Pfu68338  Pfu17439  Pfu22029 | pfu_aug1.0_11471.1_68338.t1  pfu_aug1.0_9165.1_17439.t1  pfu_aug1.0_144.1_22029.t1/  pfu_aug1.0_21598.1_54769.t1  *pfu_aug1.0_3569.1_59278.t1*  *pfu_aug1.0_9327.1_53340.t1*  *pfu_aug1.0_8721.1_24461.t1*  *pfu_aug1.0_580.1_15136.t1*  *pfu_aug1.0_52.1_50588.t1*  *pfu_aug1.0_9892.1_24638.t1*  *pfu_aug1.0_3573.1_52029.t1* |  |
| *Laternula elliptica (Lel)* | LelCALCR  LelClusterB |  | contig14182  contig11573 |
| *Pecten maximus (Pma)* | PmaCALCR |  | contig28239 |
| *Mya truncate* |  |  | Not found |
| **Gastropods** | | | |
| *Lottia gigantea (Lgi)*  www.ensemblgenomes.org | LgiG62404  LgiG238709  LgiG170937  LgiG234777  LgiG109332  LgiG134519  LgiG172709  LgiG141716 | LotgiG62404  LotgiG238709  LotgiG170937  LotgiG234777  LotgiG109332  LotgiG134519  LotgiG172709  LotgiG141716 |  |
| *Aplysia californica (Aca)*  https://blast.ncbi.nlm.nih.gov/ GCF_000002075.1 | AcaXP_005104104  AcaXP_005091164  AcaXP_005093470  AcaXP_005089839  AcaXP_005090260  AcaXP_005100091 | XP_005104104.1  XP_005091164.1  XP_005093470.1  XP_005089839.2  XP_005090260.1  XP_005100091.1 |  |
| *Biomphalaria glabrata (Bgl)*  www.vectorbase.org | BglB013441  BglB010640 | BGLB013441  BGLB010640  *BGLB007000*  *BGLB004620* |  |
| **Cephalopod** | | | |
| *Octopus bimaculoides (Obi)*  www.ensemblgenomes.org | Obi22013894  Obi22033710  Obi22036699  Obi22036695  Obi22016114  Obi22022412  Obi22027828  Obi22027591  Obi22033570  Obi22027830  Obi22016427  Obi22027368  Obi22017376 | Ocbimv22013894m  Ocbimv22033710m  Ocbimv22036699m  Ocbimv22036695m  Ocbimv22016114m  Ocbimv22022412m  Ocbimv22027828m  Ocbimv22027591m  Ocbimv22033570m  Ocbimv22027830m  Ocbimv22016427m  Ocbimv22027368m  Ocbimv22017376m  *Ocbimv22012434m*  *Ocbimv22003008m*  *Ocbimv22014169m*  *Ocbimv22036697m*  *Ocbimv22029407m/*  *Ocbimv/22029409m* |  |
| **Annelids** | | | |
| *Helobdella robusta (Hro)*  www.ensemblgenomes.org | HroG72863  HroG90909 | HelroG72863  HelroG90909  *HelroG120609*  *HelroG182402*  *HelroG165905*  *HelroG75027*  *HelroG74739*  *HelroG182403*  *HelroG135898* |  |
| *Capitella teleta (Cte)*  www.ensemblgenomes.org | CteP34226  CteP220667  CteP130410  CteP219484  CteP128185  CteP170147  CteP155191  CteP221530  CteP114577  CteP93223  CteP219483 | CapteP34226  CapteP220667  CapteP130410  CapteP219484  CapteP128185  CapteP170147  CapteP155191  CapteP221530  CapteP114577  CapteP93223  CapteP219483 |  |
| *Platynereis dumerilii (Pdu)*  https://blast.ncbi.nlm.nih.gov/Blast, (taxid:6359) | PduAKQ63005  PduAKQ63007  PduAKQ63006 | AKQ63005.1  AKQ63007.1  AKQ63006.1 |  |
| **Brachiopod** | | | |
| *Lingula anatine (Lan)*  www.ensemblgenomes.org | Lang28717  Lang21173  Lang25487  Lang31127  Lang29012  Lang19721  Lang3603  Lang19849  Lang19970  Lang19848  Lang23556  Lang11486  Lang6722  Lang32124  Lang10840 | Lan.g28717  Lan.g21173  Lang25487  Lan.g31127/g31128  Lan.g29012  Lan.g19721  Lan.g3603  Lan.g19849  Lan.g19970  Lan.g19848  Lan.g23556  Lan.g11486  Lan.g6722  Lan.g32124  Lan.g10840 |  |
| **Nematode** | | | |
| *Caenorhabditis elegans (Cel)*  www.ensemblgenomes.org | CelPdfr1  CelSeb3  CelSeb2 | WBGene00015735  WBGene00007664  WBGene00014035 |  |
| **Arthropod** | | | |
| *Daphnia pulex (Dpu)*  www.ensemblgenomes.org | Dpu299719  Dpu299721  Dpu299720  Dpu299723  Dpu62157 | DAPPUDRAFT_299719  DAPPUDRAFT_299721  DAPPUDRAFT_299720  DAPPUDRAFT_299723  DAPPUDRAFT_62157 |  |
| *Drosophila melanogaster (Dme)*  www.ensemblgenomes.org | DmeDH44R2  DmeDH44R1  DmePDFR  DmeHecR  DmeDH31R | FBgn0033744  FBgn0033932  FBgn0260753  FBgn0030437  FBgn0052843 |  |
| *Tribolium castaneum (Tca)*  www.ensemblgenomes.org | TcaTC001222  TcaTC008110  TcaTC010267  TcaTC013682  TcaTC013321  TcaTC032502  TcaTC034462  TcaTC007104 | TC001222  TC008110  TC010267  TC013682  TC013321  TC032502  TC034462  TC007104 |  |
| **DEUTEROSTOMES** | | | |
| **Echinoderm** | | | |
| *Strongylocentrotus purpuratus (Spu)*  https://blast.ncbi.nlm.nih.gov/Blast, (taxid:7668) | SpuXP_011668695  SpuXP_001200401  SpuXP_003725174  SpuXP_011668704 SpuXP_003730162  SpuXP_011663881 | XP_011668695.1  XP_001200401.3  XP_003725174.1  XP_011668704.1  XP_003730162.2  XP_011663881.1 |  |
| **Cephalochordate** | | | |
| *Branchiostoma floridae (Bfl)*  https://blast.ncbi.nlm.nih.gov/Blast, (taxid:7739) | BflBAU51796  BflXP_002599445  BflXP_002599448  BflXP_002598541  BflXP_002598604  BflXP_002587319  BflXP_002587315  BflXP_002586884  BflXP_002608820 | BAU51796.1  XP_002599445.1  XP_002599448.1  XP_002598541.1  XP_002598604.1  XP_002587319.1  XP_002587315.1  XP_002586884.1  XP_002608820.1  *XP_002587041.1*  *XP_002599449.1* |  |
| **Urochordate** | | | |
| *Ciona intestinalis (Cin)*  https://blast.ncbi.nlm.nih.gov/Blast, taxid:7719 | Cin6559  Cin6557  Cin6282  Cin2669  CinNP_001265900 | ENSCING00000006559  ENSCING00000006557  ENSCING00000006282  ENSCING00000002669  NP_001265900  *ENSCING00000020480* |  |
| **Vertebrate** | | | |
| *Homo sapiens (Hsa)* | HsaCALCR  HsaCALCRL  HsaGLP1R  HsaGIPR  HsaGCGR  HsaGLP2R  HsaGHRHR  HsaVIPR2  HsaPAC1R  HsaVIPR1  HsaPTH1R  HsaPTH2R  HsaCRHR1  HsaCRHR2 | NP_001733.1  NP_005786.1  NP_002053.3  NP_000155.1  NP_000151.1  NP_004237.1  NP_000814.2  NP_003373.2  NP_001109.2  NP_004615.2  NP_000307.1  NP_005039.1  NP_004373.2  NP_001874.2 |  |
| *Lepisosteus oculatus (Loc)*  www.ensembl.org | LocGipr  LocGlpr1  LocGlpr2  LocGhrhr1a  LocGhrhr1b  LocVipr2  LocPac1r  LocVipr1  LocPth1R  LocPth2R  LocCalcr-like  LocCalcr  LocCalcrl  LocCrhr1  LocCrhr22 | ENSLOCG00000014786  ENSLOCG00000013910  ENSLOCG00000011822  ENSLOCG00000009533  ENSLOCG00000009570  ENSLOCG00000012346  ENSLOCG00000009545  ENSLOCG00000009610  ENSLOCG00000009481  ENSLOCG00000001855  ENSLOCG00000005174  ENSLOCG00000010787  ENSLOCG00000006075  ENSLOCG00000013245  ENSLOCG00000009503 |  |

**Supplementary Table 2**

|  | Human | | Mediterranean  mussel (Mga) | Hard-shelled mussel (Mco) | | | | |
| --- | --- | --- | --- | --- | --- | --- | --- | --- |
| **Type I** | CALCRL | CALCR | CALCRIa | CALCRIa | CALCRIc | CALCRIIa | CALCRIIb | CALCRIIc |
| MgaCALCRIa | 27/46% | 29/46% | 100%/100% | 94/98% | 40/60% | 32/53% | 34/53% | 32/51% |
| McoCALCRIa | 28/46% | 30/46% | 94/98% | 100%/100% | 39/60% | 32/53% | 33/52% | 31/52% |
| McoCALCRIb | 27/44% | 28/44% | 40/60% | 39/60% | 100%/100% | 32/50% | 32/50% | 31/51% |
| **Type II** |  |  |  |  |  |  |  |  |
| McoCALCRIIa | 30/49% | 29/48% | 32/53% | 32/53% | 32/50% | 100%/100% | 47/65% | 48/69% |
| McoCALCRIIb | 31/50% | 28/49% | 34/53% | 33/52% | 32/50% | 47/65% | 100%/100% | 55/73% |
| McoCALCRIIc | 31/49% | 28/48% | 32/51% | 31/52% | 31/51% | 48/69% | 55/73% | 100%/100% |
|  |  |  |  |  |  |  |  |  |

**Supplementary Table 3**

|  | Mediterranean mussel (*Mytilus galloprovincialis*) | | | |
| --- | --- | --- | --- | --- |
|  | Precursor I | | Precursor II | |
|  | CALCIa | CALCIb | CALCIIa | CALCIIb |
| hard-shelled mussel  (*Mytilus coruscus*) |  |  |  |  |
| CALCIa | **96%** | 21% | 23% | 28% |
| CALCIb | 21% | **100%** | 23% | 41% |
| CALCIIa | 23% | 23% | **100%** | 23% |
| CALCIIb | 28% | 41% | 23% | **100%** |
| California mussel  (*Mytilus californianus*) |  |  |  |  |
| ES407016.1a | 23% | 23% | **100%** | 23% |
| ES407016.1b | 28% | 41% | 23% | **100%** |
|  |  |  |  |  |
| Pacific oyster  (*Crassostrea gigas*) |  |  |  |  |
| XP_011456505.1a | 26% | 20% | 13% | 23% |
| XP_011456505.1b | 25% | 38% | 17% | **54%** |
| XP_011441375.1 | 18% | **87%** | 17% | 38% |
|  |  |  |  |  |
| owl limpet  (*Lottia gigantea*) |  |  |  |  |
| XP_009043607a | 23% | 18% | 20% | 18% |
| XP_009043607b | 34% | **48%** | 20% | 18% |
| XP_009062318 | 28% | 48% | 20% | **74%** |
|  |  |  |  |  |
| sea hare  (*Aplysia californica*) |  |  |  |  |
| XP_012942527.1 | 31% | 51% | 23% | **67%** |
| XP_012943319.1 | 34% | **51%** | 23% | 45% |
|  |  |  |  |  |
| polychaete worm  (*Capitella teleta*) |  |  |  |  |
| G222382a | 33% | 30% | 20% | **36%** |
| G222382b  G222382c | **21%**  25% | 18%  **58%** | 13%  17% | 13%  45% |
|  |  |  |  |  |

**Supplementary Figure 1**

**Supplementary Figure 2**

MOLLUSCS

bivalves

Mediterranean mussel (*Mytilus galloprovincialis*, Mga)

>Mga.CALCI

MHQCLWRCIYLIIVLQLIEHLRADQREEDARRLMESLNQAQNDISSMMSTVDGLDNYLTTQKKR**ACNLGLNSHHCALADLDNQLQSREWLSNGHSP**GKRTTLKEEHKEKLSEVEIKRLIEDIVRKRLDLKNIRTLLEDANGSIIENSKR**TCTVELGGACRTEWASSIADQYYYLMSPHSP**GRKRRSFRKRMSPLRLFHKRLQQIKKRHSNN

>Mga.CALCII

MVETYMWLLITAILFLTTSAAVEDDVTNRGKQLIQQLDDVRHNLKSVTESLEKLSDVIEEE**CTWGGGMSDEMCSTVDIDEIQRSFQVIHDRNSP**GKRSVPSMDPSLRQFLQDLLRKKEMIQRLKHILTYADNAVHEERKK**SCQLNLGFHCQTQEYSAIADMFNFLGSGRSP**GKRRRRSIQQILKTKETL

Hard-shelled mussel (*Mytilus coruscus*, Mco)

>Mco.CALCI

MHQCLWRCIYLIIVLQLIEHLRADQREEDARRLMESLNQAQNDISSMMSTVDGLDNYLTTQKKR**ACNLGLNSHHCALADLDDQLQSREWLSNGHSP**GKRTPLQEKHKEKLSEVEIKRLIEDIVRKRLDLKNIRTLLEDANGSIIENSKR**TCTVELGGACRTEWASSIADQYYYLMSPHSP**GRKRRSFRKRMSPLRLFHKRLQQIKKRHSAN

>Mco.CALCII

MVETYMWLLITAILFLTTSAAVEDDVRNRGKQLIQQLDDVRHNLKTVTESLEKLSDVIEEE**CTWGGGMSDEMCSTVDIDEIQRSFQVIHDRNS**PGKRSVPSMDPSLRQFLQDLLRKKEMIQRLKHILTYADNAVHEERKK**SCQLNLGFHCQTQEYSAIADMFNFLGSGRSP**GKRRRRSIQQILKTKQTL

California mussel (*Mytilus californianus*, Mca)

>Mca.CALCII (ES407016.1)

MVETYMWLLITAILFLTTSAAVEDDVRNRGKQLIQQLDDVRHNLKTVTESLEKLSDVIEEEC**TWGGGMSDEMCSTVDIDEIQRSFQVIHDRNSP**GKRSVPSMDPSLRQFLQDLLRKKEMIQRLKHILTYADNAVHEERKK**SCQLNLGFHCQTQEYSAIADMFNFLGSGRSP**GKRRRRSIQQIPKTKQTS

Pacific oyster (*Crassostrea gigas*, Cgi)

>Cgi.CALCI (XP_011441375.1)

MYHWSNCWTFLIYIIFTLAPSLGNSDRADAFDEMIAAFATDTRHIRSAISSAEEVKRETQEEQIKLCRGMGPNNHPCGLTSFDVRNVRRGDDGDIEVETLNRGTRSLEGSRPDNPPTEKERMAYIAKLAKRRQDMDRILELLNEKENNIRQIRKR**TCAVELGGACRTEWASAIADQYYYLMGPHGP**GKRRRRSLINVLTGKLSHISTSNKGH

>Cgi.CALCII (XP_011456505.1)

MEGLKTSICSLLFFIVATTSLEIKSQHIREKHLVDQLDKVREQLKDLSHSVDTLRTFTQRE**ACALSLNVDICTEKYIEETADHQSKLQNLIEGNP**GKRRPKRAADMSLDLFVEDLLRKNAALQSIQQILNNMSKTVHQEKKR**SCTLNLAYHCQTSEYAGLTDLYNYLNSNASP**GKRR

eastern oyster (*Crassostrea virginica*, Cvi)

>Cvi.CALCI (MWPT03000005.1)

RKR**TCAVELGGTCRTEWASAIADQYYYLLGPHGP**GKRRRRSLIHVLQGKISH

>Cvi.CALCII (XP_022316070.1) MEGLKTSSLCSLLFFVVATTSLEIKTQHFREKHLVDQLDKVREKLQDLSASVDTLRTFTQRE**ACALSLNVDICTEKFIEGTADRQSDLHSLVEGNP**GKRRQKRRADESLDLFVEDLMRKNSALQSIQQILNNVSQKVHQEKKR**SCTLNLAYHCQTSEYAGLTDLYNYLNSNASP**GKRR

Akoya pearl oyster (*Pinctata fucata*, Pfu)

>Pfu.CALC (pfu.aug2.0_130.1_00297.t1)

MVVGNSEINLNDERLPAEVQRALAQLESEARREIRSMRDTVQNLDNDLRERQKR**QCVTGIGGHHCAVADFDRLWQSRESLNSGYGA**GKRSDDGQVETEKQIQPSEKEVLDIISSIAHRRKDLAKIHALLNDEDNRIIQHSKR**TCRIELGGACRTEWASALADQYYYLMSPHSP**GKRRRRSPSSVMKMRMAALLKSKKLMPSVI

Akoya pearl oyster (*Pinctada martensii*, Pma)

>Pma.CALC (NIJJ01057272.1)

FRKR**SCRFNLGYDCKTSEYSAITDLHNYLQSDRSP**GKRKR

Yesso scallop (*Mizuhopecten yessoensis*, Mye)

>Mye.CALC (XP_021360923.1)

MRFFILRTVYTRMVDACACLIFASFLGLFASAHAQDLHKGTRALVSNLDDVSVELVDAVRAVRRLKK**FLNDEAPSCLVSATDCSMGYIDPIEGFIDVVSNPNSP**GKRSVRSVENRTLLQKRYVNDILNKRELIGRMQSTLTDILNVIHNERKR**SCKLNLGFHCQTEEYSAIADMYNFLQSAMSP**GKR

>Mye.CALC (XP_021360924.1)

MVDACACLIFASFLGLFASAHAQDLHKGTRALVSNLDDVSVELVDAVRAVRRLKK**FLNDEAPSCLVSATDCSMGYIDPIEGFIDVVSNPNSP**GKRSVRSVENRTLLQKRYVNDILNKRELIGRMQSTLTDILNVIHNERKR**SCKLNLGFHCQTEEYSAIADMYNFLQSAMSP**GKR

>Mye.CALC (NEDP02076634.1)

RKR**TCAVEVGGTCRTEWASSIADQYYYLLGPHSP**GRRRRRSPIRLFRRKIA

Deep-sea mussel (*Bathymodiolus platifrons*, Bpl)

>Bpl.CALC (MJUT01040875.1)

KR**TCTVELGGACRTEWASSIADQYYYLMSPNSP**G

>Bpl.MJUT01050092.1

FLEDLLRKKEMIQRLKQVLSYADNTVHEERRKK**SCRLNLGFHCQTQEYSAIADMFNFLGSGRSP**GKRR

Philippine horse mussel (*Modiolus philippinarum*, Mph)

>Mph.CALC (MJUU01021507.1)

KR**TCAVELGGACRTEWASSIADQYYYLMSPHSP**G

>Mph.CALC (MJUU01073610.1)

KFRKK**SCQLNLGFHCQTQEYSAIADMFNFLGSGRSP**GKRRRRMAVRQL

>Mph.CALC (MJUU01053271.1)

KFRKK**SCQLNLGFHCQTQEYSAIADMFNFLGSGRSP**GKRRRRMAVRQL

gastropods

Owl limpet (*Lottia gigantea*, Lgi)

>Lgi.CALCI (XP_009043607/LotgiG151867)

MASLQYNYVWFLHGNNTRIYKILFKGSGVGFAEGTEKTESRMISTTPLYVTVSICLFYLTTTVYCRTDFRFLMKRDTRNNINTDSLQETLKNLDDEYQRLQKR**TCAFGINSHQCTLTSLNNKMMSQAWLSDGMSP**GKRSDLPNLTPDGRTQRLLDEMSNTKALLTVRDILTQADSPTARKR**SCSLRLGGMCLTENLNAAANQYEYLSSGLSP**GRKRRSLRHILLNRKH

>Lgi.CALCII (XP_009062318/LotgiG166645)

MGASILLGLTILLSYVNQVHLQMFHPRDISRGGQQQEEQLRRGYLIQILKSFLNRRQEDATEKRKR**SCNLNLGFHCQTDEYSSIADMYDFLQSALSP**GKRKRNVKIVSIEGS

California sea hare (*Aplysia californica*, Aca)

>Aca.CALCI (XP_012943319.1)

MSGPAATLVFLLVSLLSLNSQLTQVAGARSHDAGIYRHATREDVLGTSDALESEMLGHPKRECRFITCVFWDRIDRRNSGVTEDVISEDYEGPQGSVLRNLQEQKRAINTLRKLTEILKPDQQQIVTTRKR**TCRLRLGGHCLTEELDKAAKQYAYLKSGKSP**GRRRRETGNAAILENHDRYE

>Aca.CALCII (XP_012942527.1)

MEFVACLAEVSFLLVLVSQSAFSEGSPQRHVSLRTNLIKRMDGIQSQLADVTSNLMDLDSDLEKALYECLDIRSDLCEAIKIEAGLKDMGSLGPYWMAGKRSRDPQVNKYAGLLQELRRKRAVLKRLMKVFHGADNVLRSERKR**SCTLNLGFHCQTEEISNFADMYDFLSSPLSP**GKKRSVRAVA

Tribble's cone (*Conus tribblei*, Ctr)

>Ctr.CALC (LFLW010936661.1)

RKK**DCAFSLGNHCLTDSMDAAASEYGYLQSGNSP**G

>Ctr.CALC (LFLW010104828.1)

KW**MTCNLNLGFTCQTQEYSNIADYYDFLNSADSP**GKKR

>Ctr.CALC (LFLW010135634.1)

KW**STCNLNLGFTCQTEEYNEIANYFDYLNSANSP**GKKR

Great pond snail (*Lymnaea stagnalis*, Lst)

>Lst.CALC (FCFB01081550.1)

RKR**SCNLNLGFHCQTEEIANFADVYDFLSSPHSP**GKKRRKRS**CNLNLGFHCQTEEIANFADYDFLSSPHSP**GKKR

>Lst.CALC (FCFB01003250.1)

RDLWKR**ACRFNLGGHCLTEEMNRAAEIYYYLKSPYSP**GKR**ACRFNLGGHCLTEEMNRAAEIYYYLKSPYSP**G

Freshwater snail (*Biomphalaria glabrata*, Bgl)

>Bgl.CALC (APKA01053835.1)

RKR**ACNLNLGFHCQTEDISNFADMYDYLSSPHSP**GKKR

>Bgl.CALC (APKA01024881.1)

**CRINLGNHCLTEEMDNLADSYHYLMSSNSP**GRKRR**ACRINLGNHCLTEEMDNLADSYHYLMSSNSP**GRKR

Big-ear radix (*Radix auricularia*, Rau)

>Rau.CALC (MUZC01004631.1)

RKRS**CNLNLGFHCQTEEIANFADMYDFLSSPLSP**GKKR

>Rau.CALC (MUZC01003141.1)

KR**ACRFNLGGHCLTEEMNRAAEIYYYLKSPDSP**G

Vampire snail (*Colubraria reticulata*, Cre)

>Cre.CALCR (CVMW01072519.1)

ESRVVDLDALKENLASIEENLSDFQKR**SCAVGLNSHHCAIAHLDDVMLGSDYLSGGFSP**GKRDTELPEDVRSQAEILLQNLSEQKHAVRTLRTLLGKLGVRVKQQMKR**TCAFRLGNHCLTEALDHAANQYYYLQSPASP**GRRR

cephalopod

California two-spot octopus (*Octopus bimaculoides*, Obi)

>Obi.CALC (LGKD01399119.1)

KK**GCTTNFEHLCSAQEAAELAELLRFLQSGEGP**GRK

**ANNELIDS**

Polychaete worm (*Capitella teleta*, Cte)

>Cte.CALC (CapteG222382)

MDHVNDLHQSNQFLLLRDDALLPENTQIRWGKVVKQQGGLYVDQDDAGWCASREQRCILL

LCLGKPLTVYQNLGETRKGLTALKRVVSDLDGDILQKQKR**TCQVGGGMNRHCTAALLNSK**

**MEMANWLNSGMSP**GKKRSLEEVPRTVSKRKR**LSPCDVSGGNAGYSCYLDGLATVSKNRDW**

**LDTNPLAP**GKRSSSTHDRSRNQSNSVHSSDFHDLARKRKGLDILRRILSEMEADLIYEQK

R**TCQFNLGGHCATESAASVADHWHYLNSPLSP**GRKRRDTGLYKAVVSGRIFKDSKH

**Supplementary Figure 3**

**Supplementary Figure 4**

**Supplementary Figure 5**

**Supplementary Figure 6**

**Supplementary Figure 7**

**Supplementary Data 1**

*Mytilus galloprovincialis*

>Mga.CALCRIa

MSDLSDNPHLKALLESSLSCVKTPDDDDLYCNTTSDFFGCWNFTRAGTAMTKPCPYLPGFNTKEIAYKNCSENGTWERANYMKCRMTREIEEDLNKEVLNAFRHIYISLVGFSISITLLVISLFIFFKFRQLRCDRITLHKNLFISYLLTAIMYILYNVLVIMDGDVLHDQPIWCVVLHVVTQYCVVSNFAWMFCEGVYLHTIMVKTFITGKWLIIVCTVIGWVCPFVLVGVYTAVRASSKDDNILCWHQESTLQWIMYAPVVASIFLNLIFLCNIVRLLITKLRQIPEAGQTRKATRATLILIPLLGIQYLMFPIKPAEGSNWEEVYLIVVALHLSLQGAFVSIIFCFCNGEVITVIKRKWALHRESTFTSKGRYRAPSTIGTTSYTLVDHMSGVATSVT

>Mga.CALCRIb

SGNLYCNATTDTLGGCWNITRAGQSAKIPCPELMESSSYGGSAYLNCTENGIWNTINGSIRGDYTHCQFWFWDAGKGDHLPVYVFITGNVVSIILLSVALTIFLKFRFRQLKCGRIFIHKNLFLSYILTGLTWILYNSLVVLNSDALEHNYVWC

> Mga.CALCRIc

MTPEETLSFFETVSEEQTKCLERISKIKPNFGDNLFCNGTAGRMGACWNHTLAGETAVQSRILAINSSDRISYHCTENGTWDIRGENYTHLNFFVFRQLRCGRVLIHKNLFLSYVFTGLTWILYYKLVVLDGDIIDANPQWWCQFLHVLAQYFMQCNFAWMFCEGLYLHTVLIRVFSNGKILTFICYAIGWGCWMNESSLQWIMYGPIVISLAVSVNIIFLINIVRLLMTKLKDLPEAAQNKTRKAARATLVLIPLLGFQYLLLPMRPDHGSPFEDVYSYCSAILTSCQKRKWDQHWLMYGSGRRKHRCSSTHYTVTENMADDCNKK

>Mga.CALCRIIa

MVSLMLALCIFCCFRQLKCTRVSIHKNMFVSYILAAILWMSYYLSSALHPDVLQENPVWCRLLHILAQYATSSNYAWMFCEGLYLHTVLILAFINEKHVLILCIITGWVVPMIQTVIYFAIRVTSSEINKLCWHGQTNLQWIFLGPIALWLALNTFFLVNILRILCTKVSSMRQSNPLRHTLRATLILIPLLGIQSVAIPFRPVGHEDFLYAYNIVSAILVSFQGFFVSLLFCFFNGEVILLLKNQLGVHRQRKMSRSDTARSLMYFQLSNNNNASRPRLSICSPVVKTNDETGVKLVTDTKPDEKPPDQFIESNV

>Mga.CALCRIIb

MAARNIRSSENLSFDQIMLKEIFINFIKCNETVLQEPYPNDQLYCNATFDGYTCWNHTKAGTKTYGNCPLFMVEKFGQQPGGQPFKECNTDGTWYKHPHTQRSWTNYAPCSDTIKVQQRANNVLYAYFSGYGFRQLKCTRVTIHKHLFVSYILTAVFWITYYATTSFDPHVVADNPVINVIFLMNIIRILCSKVNANRPQSLQSLRRQSLKATCILIPLLGIQYIALPFRPNDGNEHGQYVYDMVSACLSSFQVFQGFLVSLLFCFLNGEV

>Mga.CALCRIIc

MTHNGTISDTNQLSFQELMMNEIWQNFMKCNETVLHKSYPSDGGYLYCNATFDGYDCWDYARAGTRTYGTCPDFLKHQFGHHVGMGLPYKDCNIDGTWYRHPDSDRVWTNYSPCSDTEKVRRQLKCTRVTIHKHLFISYIITALLWISYYATSSFDPQVYINNPVVWCRILHVLAQYATVCNYAWMFCEGFYLHTILVMTFTREKILLIICYLTGWGGIPVIPSAIYSGLRSTNPVIDKKFRQSLRATLILIPLLGVQCFAIPLRPDESDEYGQYIYDMISAFLASFQFRQSLKATCILIPLLGIQYIALPFRPNDGNEHGQYVYDMVSAFQGFLVSLLFCFLNGEVQVLFLMKNQYVLVRRRISRTETTGKSFIYSAVQQADANANRKKSIQLANQKIYCKSEFTGTTQPQQSE

>Mga.PDFR

MVKFDKYPSTLMECLDSQGDALTVPSNLFCNATWDTILCWPPTLIGSITKLPCPGKHDTGMERQSYASKECGLDGNWIGIHPGKEIFPEEFEPGWTNYSECIGVIYETVKNTSDNIMNVSESASVSVGALSIPHNGADIFGAALLVLSLILILASLGITCCNNAIQSTRTRFYRNLFAAFIFHDVLELVVRIGRVFHSDAIIRDIETCVSFGVLLTLLSTAIFSWFLMIGISFMLTFKGVVVENRMYYVMCLLGWFMPTVVTITWLSVTVLGGNLSCWDGELYIARMTSSFWIIQGSIVIFLLFTWLCILSFLLRYKEWEMHRKYSENKDLAVELTNIKQSGYKMFATMCFMTGAYIIYLSCSQSPLTESLSYLLVLVMFSRGIIVAVFMCFLEEHCKCWDGVYAVEGSTTTDSESLSSRNSSHPSHYGHHTVLPNHGIISM

> Mga.CRHR MDYEHMMMEFEGRAPYKLACLQRQQQYHETYVDNGNSYCNMTWDGLSCWPASLVGEFAIIQCPQNMQGVDSTQNATRLCTTNGTWALKSDYNSCLTDTNPKDLYNGVESVHEYSRRIIYNLGFIVSTITLIIALFIFLYFRSLRCLRNIIHSHLIATFILRNLLWIMMQHTLWPIVQADEKWACKLEVALFNYAQMTNFFWMLVEGLYLHIIIVWTYSADKIRKWYFIIIGWCVPAVIITIWVIFRTQVTADETSDVAACFMPKSKNYAESEADKLDYIYIAPILFVLAVNIIFLGSIIWILVTKLRSSHSLETKQLRKAVKATITLFPLLGITYVIFIWPPSDNHIFVEVHQYINAFLQSFQGFFVALFYCFLNGEVKTVLKKKLKIFQDTRALSTRYTKTSMVQDMSVVGRDSIAPTNGKNTSGRNGFRFMSNSQRFNDDEQAESENML

>Mga.ClusterAIa

MIKNRIVNRFFLCWYFSQVTVVYSTLERKCHSRLGYNNPDVFELFSCAWCYEHLFSIKPQRQLYASDRVALLYNPLKKLLISPDINNQTIINEVCSTVDNDECHRWKSCCVAANDCCRRQLSTPLGDKNETCGRTWDGWGCWDDTEPDKRVYLSCPLYLQYSLPIRQAVKTCHHNASWERRDGREWTNYQPCLKVDDLKTSIFVGLGCSICSILVLFPAIFIFIRYQGLRKQHRIRLHINLFLSLLLKEIAVACWDMLVTYERMTNIDTSSTVLAGNGAGCKILSFVKIYFKCASFTWMFCEGLFLHRLMSDAFSPPRSLIPFYIAGWTVPFTTTLIYAILRAVLANESCWSTSYGHFEWIFDVPNLIFLVVNLIFLCNILRILLTQMQSHPNEPSNFRKALKAMFVLIPLFGIQLVVTIYRVPITEPGGLQYERFSIVINNLQGFLVALIFCFLNNEVFSHLRRTWRRRIRDRGISHSRSKFNSTSINMTLTSRAEGDFQCVHRSLCDKVEENGGDHLSRDINEQIPCENFKDDSLDNSCNRVLLSNNDT

>Mga.ClusterAIb

MYKAPGWRLLEILYALQLMFECVCTLERTCNSRIGIHKQKEFEYFSCAWCYDFLFQTTHKTLIASDVVHFLYHTIEDRIILPDINNQTDVNDICSTLDKDECHRWQTCCSAASACCQKQLSVPLGDLNNTCDRTWDGWICWDDTHPGQHVYQSCPLYLTFSVPSRQAVKTCLSNATWERREGFEWTDYQTCLYVDDLKTSIYIGFACSLVSIAALFPAVVIFIRYQALRKQHRIRLHINFFMSLLLKESAVALWDMLVTFERITNSDSSSTVLAQNGAGCKTLSFLKIYAKAASFTWMFCEGYYLHRLMSDAFSPPRSLIPIFAAGWAIPFVSTLIYAILRIVYANESCWSTSYGHFEWIFDAPNLACLLVNLIFLCNILRILLTQIQSHPNEPSHFRRAVKATFVLIPLFGVQLFVTIYRIPTSSPGGLEYERFSIVTNNLQGFFVALIFCFLNNEVISNIRRSWRRRVRDRGASHSRRTNMTSINMSLTSRTDADFHHVNKHVCDIKNENGGCSPDKGDNMFCPYDENSVKSVPKTDFSNL

>Mga.ClusterAII

MEGYCQTRFHYLNQKLFNLLTCSMCYQYLFPKNKELRSKGLFLEATSESRYKEGTQILANISNSTFVRNVCKTLEAEGDCARWTDCCNSAIKCCKDQLAAPFLYNGTSHCPRIWDGYGCFGDTLPGVREYIQCPSYIEHGVTHANAFKDCTENGSWYVDPLTNKTWTDYTSCVPIEHPMILVYMSLACNIISLLLLVPSCAIFLAFKQLRNQHRIKLHICFFTSFILVSVVALMWDFLVHSNRLTTSADSTLQKNTSGCKLLNALLRYTQTSNYFWMFCEGFYLHRLIVHAFKVPKGLLGYYVMGWGIAWVPVVIYCIIRATDEDLDERCWVTDAGHYEWIIFIPNVLCLFMNVVFFVNILRILLTQLQSHPNEPSNYRRALKATFVLIPLFGIQWGFVIHRPGFTLWYEVVRIIVQYTQGAIVSLVFCIFNGEVHSHLKSCLRKKWPSAFRDDPGRFQSTVSGTQYSHVSSGRRGTQQTDHYIPLNTVPDDQSKQNGHVG

>Mga.ClusterBI

MMTGNTNSTAPRMTEQMKKQLMRLHVKKQTCEAKIINYTIPQTGSFCDMVWDNIMCWDATPAGTTAKMKCPHYIEGFSNTEFATRTCTENGTWYVSPHTNHTWTDYTRCPTVNLSLMEEHFPRIKLMYNIGYGLSLGSLLVAVFLMCFCRRLYSKSNTLHINLFFAFILRASMSCIRDILFVDGFGLSKDVKRGTNGGVTFIQEGSHWECKLIYCLLIYGVTVTCTWIFTEALYLHMLVYKTLFTERHGVKLYMIIGWFTPLLFVIPWIIVRIELEDVYCWNWTGNTDYQWIITGPVLLINGINFIFFVDLVRVLYKRVHTNKRVTGSRKIRKLSKFIVVLIPLFGVLYIVFSFLYNPKLNEERDVVVMYAEMFYNSFQGLLLAIVFCFLNEEIHVEIRKCWYKYALSRTDSTMYTRTAMLSTWRHTGSQSSRGHSNIDNTQSDICLNDNVGGRNYRNGVKKPKSKMVPNVRIRFQSSDVRNSRSSSNSSPTQDRESRYLKLEEAYITRT

>Mga.ClusterBII

MRSRMQFRKSHALYVVLMYTMLDNGKVKGSDLLINNEEQQRKVLLLEELQCNKSMQQEKQTEGLYCNITWDGISCWPATKAGTVAVKPCPDYINRLDPTENVTRRCESDGSWFVIADTNKTWSDYSACVMNDIPVNGVPGQPVPAIIKDHMDNLTIMKHTGYGLSLASLLVAVIIMLYFKKLHCPRNTIHLNLFLSFILRAIISFIKNGIMIEGLGFSSDSY

>Mga.ClusterBIV

MIYFRKLHCQRNTVHINMFISFILRSIICFIKDIDITPEVYYSSTIEGETSFGQAWICKSVYVIFYYVLTANFMWIFVEGLFLHTFVLSTKYNVSRHIYRTFLILGWCVPLLSVIPWVIVRYMFENTLCWNTMDNRFFWIIKGPIMVTCIINLVFFINIIRVLYTKLNAAHTKDPNKYRKLARSTLVLIPLFGVYYAVFIAVPICMDPNLEVIWMYSEMFFNSFQGFAVALLFCFMNGEVQREIKKHWRRRRIMRRQSNMSSRSKTYAIDHDTHHTCVQDTWDSNGNMDLKELTIPVCNVDLNVNSPQELDENLPILND

*Mytilus coruscus*

>Mco. CALCRIa

MSDLTNNPHLKAFLESSLSCVKTPDDGNLYCNTTSDILGCWNFTKAGTAMIKQCPYLPGFNTKEMAHKNCSENGTWERANYMECRMTREIEEDMNKEVLNAFRHIYISLVGFSISITLLVISLFIFFKFRQLRCDRITLHKNLFISYLLTAIMYILYNVLVIMNGDVLHDQPIWCVVLHVITQYCVVCNFAWMFCEGVYLHTIMVKTFTTGKWLIIVCTVIGWVCPFVLVGVYAAVRASSKDDNILCWNQESTLQWIMFAPVVASIFLNLIFLCNIVRLLITKLRQIPEAGQTRKATRATLILIPLLGIQYLLFPIKPAEGSSWEEVYLIVVALHLSLQGAFVSIIFCFCNGEVITVIKRKWALHRESTFTSKGRYRAPSTIGTTSYTLVDHMSGVATSVT

>Mco. CALCRIb

MTPEEKLLLYETISKEQTKCIESISKIRPNFGDSLFCNGTAGRLGACWNHTLAGETAVQSRIIGIDSYRISYHCTENGTWDNRGENYTLLNDNSDESQGYVYVYIAGNALSLVLLTIALFIFFGFRQLRCGRVLIHKNLFLSYVFTGLTWILYYKLVILDGDIIHANPWWCQFLHVLAQYFMQCNFAWMFCEGLYLHTVLTRVFSNGKILTFICYAIGWGYPLIPTIIYTVLRSRTGKRCWHEESSLQWIMYGPIVVSICINVMFLVNIVRLLMTKLQKIPEASQSKKAARATLVLLPLLGLQYLVLPMRPSENSDFTDIYLYSVAVLTSLQGSFVSIMYCFCNSEITAILKRKWDQHWLMYGSGRRKHRCSSTHYTVTENMADDCHKKFTDKPEMVPLQETVCDV

>Mco. CALCRIc

TLVQGGENHSGNLYCNATTDTLGGCWNITRAGQSAKIPCPELMESSSYGSAYLNCTENGIWNTINGSIRGDYTHCQFWDAGKGDHLPVYVFITGNVVSIILLSVALTIFLKFRQLKCGRIFIHKNLFLSYILTGLTWILYNSLVVLNSDALEHNYVWC

>Mco. CALCRIIa

MENGTSKNDLSFDEIYYWSIKKAKTYCDQTVLKQEYPGDGNLYCNATFDGYSCWNYTKAGTRAYGECPQFFIYEFGHTDSLPFKDCNKDGTWFRHPETNTTWSDYTTCAETIKARRAHTVLYVYFSGYVLSMVSLVLALCIFCCFRQLKCTRVSIHKNMFVSYILAAILWMSYYLSSSLHPDVLNENPIWCRLLHILAQYATSSNYAWMFCEGLYLHTILILAFINEKHVLILCIITGWVVPMIQTIIYFAIRVTSSEINKLCWHGQTNLQWIFLGPIALWLALNTFFLVNILRILCTKVSSMRQSNPLRHTLKATLILIPLLGIQFVAIPFRPVGHEDFLYAYNLLSAILVSFQGFFVSLLFCFFNGEVILLLKSQLGVRKQRKMSRSDTARSSMYIQLSNNNNASRPKSSICSAVVKTNDETCVKLVTDTKLDENPPDDFIESNV

>Mco.CALCRIIb

MAADRNILSSESLSFDQIMQREIFVNFIKCNETVLQKPYPIDEQLYCNATFDGYTCWNYTKAGTKTYGYCPLFMVEEFGQQPGQPFKECNTDGTWYKHPHTQRSWTNYAPCSDTMKVQRANNVLYAYFSGYGVSIVVLIVALLILASFRQLKCTRVTIHKHLFVSYILTAVFWIIYYATTSFDPHVVADNPIWCRILHVLSQYVTVCNYAWMFCEGFYLHTVLVLTFTNEKTLLKICYVVGWGIPVVPSVVYTVLRSINEDFNNSCWHDDTILIWTFSGPIALSLLINVIFLMNIIRILCSKVNANRPQSLQSLRQSLKATCILIPLLGIQYIALPFRPNDGNEHGKYVYDMASACLSSFQGFLVSLLFCFLNGEVLSLLKAQIKMLQRCVSYKETLPSNMYASVHQSEGGKHITDIELK

>Mco. CALCRIIc

MAYNGTISDTEQLSFQEMMIKEIWQNFMICNETILHKPYPSDGYLYCNATFDGYDCWDYARAGTRTYGKCPEFLKHQFGHHLGLPYKDCNIDGTWYRHPDSDRVWTNYSPCSDTEKVRRAENVLFVYFSGYSLSVVCLVISLLIFTCFRQLKCTRVTIHKHLFISYIITALLWISYYATSSFDPKVYVNNPVWCRIIHVLAQYATVCNYAWMFCEGFYLHTILVMTFTREKILLIICYITGWGIPVLPSAIYSGLRSSNPVIDKNCWHKDTYLKYIFSGPIAVSLIVNFIFLINVLRILCSKVRSFNQQESNPFRQSLRATLILIPLLGVQCFAIPLRPDESDEYGQYIYDMISAFLASFQGFLVSLLFCFLNGEVLFLMKNQYVVVRRRMSRTETTGKSFIYSAVQQADANANRKKSIQLANQKIYCKPEFTGTTQPQQLEDQHYVLDQTTDV

>Mco. PDFR

MNKFENYPSTLMECLDSQGDALTVPSNLFCNATWDTILCWPPTLIGSVTKLPCPGKHETGMERQSYASKECGLDGNWIGIHPGKEIFPEEFEPGWTNYSECIDVIYEAVQNTSDNVMNVSESASVSVGALSIPHNGADIFGAALLVLSLILILASLGITCCNNAIQSTRTRFYRNLFAAFIFHDILELIVRIGRVFHSDAIIRDIETCVSFGVLLTLLSTAIFSWFLMIGISFMLTFKGVVVENRMYYVMCLLGWFMPTVVTITWLSVTVLGGNLSCWDGELYIARMTSSFWIIQGSIVIFLLFTWLCILSFLLRYKEYEMQRKYSENKDLAVELTNIKQSGYKMFATMCFVTGAYIIYLSCSQSPLTESLSYLLVLVMFSRGIIVAVFMCFLEEHCKCWDGVYAVEGSTSTD

SESLSSRNSSHPSHYGHHTILPNHGIISI

> Mco.CRHR

MDYEHMMMEFEGRAPYKLECLQRQQQYHATYVDNGNSYCNMTWDGLSCWPATLVGEFAIIQCPQDMQGVDSTQNATRFCTTNGTWALKSDYNSCLTDTNAKDLYNGVESVHEYSRRIIYNLGFIVSTITLIIALFIFLYFRSLRCLRNIIHSHLIATFILRNLLWIMMQHTLWPIVQADEKWACKLEVALFNYAQMTNFFWMLVEGLYLHIIIVWTYSADKIRKWYFIIIGWCVPAVIIAIWVILRTQVRADETSDVAACFMPKSKNYAESEADKLDYIYIAPILFVLAVNIIFLGSIIWILVTKLRSSHSLETKQLRKAVKATITLFPLLGITYVIFIWPPSDNHIFVEVHQYINAFLQSFQGFFVALFYCFLNEEVQTVIKKKITNWVESRANYSLITRLRRS

>Mco. ClusterAIa

MIKNRIVNSFFLCWYFTQLTVVYSTLERKCHSRLGFHNSHVFELFSCAWCYEYLFTIKPQRQLFASDRVPFLYNPSKKLLISPDINNQTIINEVCSTVDNDECHRWKSCCVAANNCCKRQLSVPIGDVNETCGRTWDGWGCWDDTEPDKRVYLSCPLYLQYSLPIRQAVKTCHHNASWEIRDGREWTNYQPCLTVDDLKTSIFVGLGCSISSILVLFPAIFIFIRYQGLRKQHRIRLHINLFLSLLLKEIAVACWDMLVTYERITNIDTSSTVLAGNGAGCKILSFVKIYFKCASFTWMFCEGLFLHRLMSDAFSPPRSLIPFYIAGWTVPFITTLIYAILRAVHANESCWSTSYGHFEWIFDVPNLIFLAVNLIFLCNILRILLTQMQSHPNEPSNFRKALKAMFVLIPLFGIQLVVTIYRVPITKAGGLQYERFSIIINNLQGFLVALIFCFLNNEVFSYLRRTWRRRIRDRGISHSRSKFNSTSVNMTLTSRAEGDFQCVHRSLFDKVEENGGDCISREVNEHIPCVNFKDNSLDNLCNRVLLSNNDA

>Mco. ClusterAIb

MCKIPVWRLLEILCFQLMFKCVCTVERTCNSRIGTHKQKEFEYFSCAWCYDFLFQTTHKTLIASEIVHYLYHPIENRIILPDINNQTDVDDVCSTLDKDECQRWKTCCRAASACCQKQLSISLGDLNNTCDRTWDGWICWDDTNPGQQVYQSCPLYLTFSVPSRQAVKTCLSNATWERREGFEWTDYQPCLYVDDLKTSIYIGFACSLVSIAALFPAIIIFIRYQALRKQHRIRLHINFFLSLLLKESAVALWDMLVTYERITNSDTSSTVLAQNGAGCKILSFLKIYAKGASFTWMFCEGYYLHRLMSDAFSPPRSLIPIFVAGWAIPFVSTLIYAVLRIVYANESCWSTSYGHFEWIFDAPNLACLLVNLIFLCNILRILLTQIQSHPNEPSHFRRAVKATFVLIPLFGVQLFVTIYRIPTSSPGGLEYERFSIVTNNLQGFCVALIFCFLNNEVSSHIQLQRCFNKLVISNIRRSWRRRVRDRGASHSRRTDLTSVNMSLTSRTDADFHCVNKPLCDTKNENGGSSLDKGDNMFCPYDESSMKSKHNTDISNL

>Mco.ClusterAIc

MILGIEYTSLLISIMFVRLTSSMANKKCHSRLGYHDPRTFDLYACAWCYDFLFSVNEKALSASFHEPYLRDRDQMIAGYHLVPDITDDGNFSRICSTLTNDECKRWRSCCINAHDCCGRQLSAPPVTNETCARTWDGWGCWDDTPPSTSVYLSCPAYIAFSIPTIQAEKKCLSDGTWQIRDGQPWTNYQPCLIFHDLKTSVYIGIACSALSISLLLPALIIFLKYNSLRKQCRIRLHIHFFLALLLKEIASVLWDVLVTYDKLSNDTVYTTTLAQNTHWCKVLSFCKMFMKCSTYSWMFCEGFYLHQLLSNAFSPPRSLICITVFGWGIPLLSASLYSLVRVIYSDENCWSIFFQYTEWIMYTPNLVFIMANLLFLCNIVRILLTQMQSHPNEPSNFRRAVKATFVLIPLFGVHLFVTIYRIPISKTGGMEYERFTICIDNLQGFFVALIFCFLNNEVNGKLRRTWRRQVSERFTSRNRRMTLTLNQTLSLRSDIDFKSPKNSRKDEEKTNIVNGDHKRSNNSVVDV

>Mco.ClusterAId

MEKNKNNNMHYCHCPSINNASSLIYIENSIGKKKLSKQWHQCCKDAANCCNEQTYSEDIDEMSEVRGDTCPHTWDGWMCWNKTSKKTAVHNQCPRFLSLSDYGENATKFCTGNGTWWVDPETLQERTDYSKCLSKDALKRLEFHKISTIVSLSINAFGIFLLVPAIIIFIRYRNLRRQNRIKLHINLFISLLLCGIAVMCWDVVIKYNRIVNQQDDLIQYNQDGCKVLNMIEHYFR

STTYYWMLCEGFYLHRLLLNAFVPPKRLIGYYITGWGMPLLTALIYTVLRIRLFDNSDCWIDPVSGKYHGLEWILFAPTILCIILLVADQCVFPFEHSSSIGSTAFSSQ

>Mco.ClusterAIIa

KTLTWYYITGWGGSFLPILVYVIVRLTTADYDCWVKNIGIYEWILYIPNLICILLNLIFLCSIIRIMLTRLQSHPNEPSNYRRGLKATFLLVPLFGIQLFFITYRPPAEKADRLLYEIVSKVIIDSQGSLVSLIFFYMNAEVHTALKYQLRVIHLQFPYKTKKSISQNQSATHMSSIRQTSQRNGCYNGPKFEKTVHINGDVKEYVFS

>Mco.ClusterAIIb

MEGYCQTRFHYLNQKFFNFITCSMCYQYLFPTNKELVSRGLFLEATSESRYKEGTQILANISNATFAKNVCKTLEAEGDCKRWTDCCYSAIKCCKDQLAAPFLHNGTSHCPRIWDGYGCFGDTLPGVREYIQCPSYIEHGVTHALAFKDCTENGSWYVDPETNKTWTDYRSCVPIDHPMVLVYISLACNIISLLLLVPSCAIFLAFKQLRNQHRIKLHICFFTSFILVSVVAVMWDFLVHFNRLNTSSDSTLQRNTSGCKLLNALLRYTQTSNYFWMFCEGFYLHRLIVHAFKVPKGLLGYYVMGWGIAWVPVVIYSIIRATDEDLDERCWVTDAGHYEWIIFIPNVLCLFMNVVFFVNILRILLTQLQSHPNEPSNYRRALKATFVLIPLFGIQWGFVIHRPGFTLWYEVVRVIVQYTQGAIVSLVFCIFNGEVHSHLKSCLRKKWPSAFRDDPGRFQSTVSGTQYSHVSSGRRGTQQNDHYIPLNTVPDDQLRQNGHVG

>Mco.ClusterAIIc

MSCCERQINAFPTSTTDLKCPMTWDGFGCVDSTNPGTNSIIECPDFIEYGFSSGYARKSCTVNGTWWVDSKSNREWTDYTPCLNLKIYKSLVYVGIACSSASLVLLVPACVVFLSLRQLRSQHRIRLHTCLFLSFIMTCIMTILWDLLIYNDRLDNPASTRMHQNTIFCRVLYAVHRYCSTCNYFWMFCE

>Mco. ClusterBIa

MTMYNFTDLEAWLHYKDYEKTFITIEDQKDVIVKKKLECYGLIFTEPPRTSNGLYCNITWDGISCWPATKAGTVAVKPCPDYINRLDPTENVTRRCDADGTWYVHADTNKSWSDYSACVMNNIPVNGVPGQPVPAIIKEHMDNLTIMKHTGYGLSLASLLVAVIIMLYFKKLHCPRNTIHLNLFLSFILRAIISFIKNGIMIEGLGFSSDVYYNEKGQLAFLPEGLHWECRMFMTIVNYIMAANYAWIFVEALYLQMLISVAVFTEKKHLHIYMLLGWTFPLAFIIPWVIVRSTVDNKLCWTTYDIRWVEWIIHGPILLTIVINFTIFINIVRVLFTKLNSSPCPETKKFRYRRLAKSTLVLIPLFGVHYMVFSIIFQIVETQSKGLGLLIYFYLEIFFNSYQGFILSLLFCFFNSEVQNEFKKAWRRYTLNRSGSSRWRSTFTSRTKSWKDHRNSDSNGVTSRGDRVRPNEDELHSSPTLNITQSPITQSSYAYDVKTSQEFTGQDIEICENTPWLNNNHTAYVEQHGLPEEQADENL

>Mco. ClusterBIb

MSLQLACFVLLLSTETMGVIVDITLNEQNRGIAEAGQSCQGLIENEGNQKNDSFCTRVWDTISCWPPTPSGSMVTIPCPNYLNGFNTKKFAKKFCTMNGTWFFHPSLNVTWTDYRECPYVSALEEIPELIRNHMPYIRLMFNIGYGISLVSLVLATAIMLTFRRLRCPRNIIHIHLFISFILRASISFMKENLLVNGTGFPSDIREINGNLEFITEGTHWQCKLFFTSFHYI

>Mco. ClusterBIc

MPYLYDLFEGKLKMNPVLLLTFFFFEVYGGVVDITLSEQNHGIGDANQTCTQLIEKEQNTKNDSFCERVWDTIACWPSTPPGSLATIPCPNYLNGFNTAAFAHKYCTSNGTWFYHPVLNLTWTDYTNCPYTSIMEDIPELIKKHMPYIRLVFNIGYGISLVSLVLATTLMIAFRRLRCPRNTVHINLFISFILRASISFMKENLLVNSAAFASDVKEVNGKLQFIMEGTHWQCKLFFTVFHYILATNYIWILVEGLYLNMLITVAVFSEKSGIKWFIIFGWASPLLFVGPW

>Mco. ClusterBId

NTLCWNTHNKSGYFWIMRGPIVLSVAVNFIIFLNTIRVLYTKLTAFNSFETKKFRYRRLAKSTMVLIPLFGVHYIVFIGLPDNVSKEAELVKLYFEMFFNSIQGFVVALLFCFLNGDVQTEIAKTWKRFRLSHGGGSLRSHRDTVTSYVSRGRGSIVSNSNSETERKTSNHELKPLTRIQNDANENHKTNGHVQWQDESTPMIDYQSENCVENHRPND

>Mco. ClusterBII

MMTGNTNFTVPRMSERMKTKFEQLMRLHVEEQKCEAKIINYTAPQTGAFCDMVWDNIMCWDATPAGTTAKMKCPHYVEGFSNTEFATKTCTENGTWYVSPHTNGTWTDYTRCPPVNLSLMEEHLPRIKLMYNIGYGLSLGSLLVAVFLMCFCRRLYSKSNTLHINLFFAFILRASMSCIRDILFVDGFGLSKDVKRGTNGGITFIQEGSHWECKLIYCLLLYGVTVTCTWIFTEALYLHMLVYKTLFTERHGVKLYMIIGWLTPLLFVIPWIIVRIELEDVYCWNWTGNTDYQWIITGPVLLINGVNFIFFVDLVRVLYKRVHTNKRVTGSRKIRKLSKFIVVLIPLFGVLYIVFSFLYNPKLNEERDVVVMYAEMFYNSFQGLLLAIVFCFLNEEIHVEIRKCWYKYALSRTDSTMYTRTAMLSTWRHTGSQSSRGHSNIDNTQSDICLNDNVGGRNYRNGVKKPKSKMVPNVRIRFQSSDVRNSRSSSNSSPTMDRESRYLKLEEAYITRT

>Mco. ClusterBIII

RNTVHVNLFISFILRSLVTIIRDNVLVQGLGLPGDVEQTPYDTVIFITNGTHWECKLLFTMFYYILSASYMWIFVEGLHLYILIMVSVFSERKCVRWYILLGWGLPTLSVFPWVGLRITFENTLCWNTNPTPGYFWILRGPVVLSIVVNFFFFINILWSLNSKMRKALSRSARKNKYRRLAKSTLVLIPLFGIHYIVFIGVPDDIPPVA

>Mco. ClusterBIVa

MVNILNMSNLSVFLFIILFERSSASWCSTEKEITQLDQLKALSKALTDCNASLSEPIN

NNTECSGHFDGIMCWPPTKAGEISKQSCPDYIHGFNTSGYATKECLPTGTWYFHPKFNKSFADFTGCIPNIPFPSLPKPTRIELIKSHMESIQIIYRIGYAVSLIFLVIAVVVMTIFSKLRCQRNTIHMNLFLSFIFRSIICFIKDEHGIPEDETNIMDEVLRQLNSSEASWWCKLVHALFFYILVANYMWIFVEGIYLHTLIFVTTFHNISHKMFRILIVLGWVSPIVCVMPWIIVRVIYEDTLCWNLHNQDNGFHWILQGPIIATLSVNFFFFLNIIRVLFTKLSATNTRDPKCYRKLAKSTLVLIPLFGVHYIMFMAIPICLEPEMEVAWLYIELFFSSFQGFAVSMLFCFTLDEVKSEIKKHWQRHMLRRQSMTSTRSTRTFSVQSSGSGHEQESPTYKRQNGLSLIRAVESCNSLGKDITNYDTETELYHNNSDENGGLYFESSVKLKDDNENLPLFSSEIATVL

>Mco.ClusterBIVb

MCYILCSIVLVLIFRICGAKVCHQNEVSDVSKSLQYEQEMIQKAEEKCNVSMLSASVRNDSYCNQVFDGIMCWPPTESGSLSIQPCPSYMYSLLPNGNASRTCLRNGEWYVHPIFNKTWTNLSGCFDPTLKYNSPSVVPAIVKEHMRYIQILYDVGYSISLVSLLVAVSIMLYFRNLHCQRNTIHINMFVSFIFRSIICLIKDSYVIPEINSIYSTDENKNIQSFGQSVGCKIVYTFFYYILSTNFMWIFIEGIYMHTFVMSTKYNVSSSLYKTLLVFGWGTPSTFVIPWVIMRIKHENTLCWSTNDVDKGYYWIIKGPITVTVVINFIFFVNIMRVLYTKLTASHTRNPKRYRKLAKSTLILIPLFGVYYLAFIAVPVCMEPILEVIWLYTEMFFNSFQGFAVAILFCFMNEEVRREIRKVKRDHKLRRNSHISNRSTSRTVEQFFQSEHDNIMLGAEPKPANVHFELDEKSRIILEKTKHLFNDGNDRNLNTTVNGIYLNGENSNLVSINDTTIES

>Mco. ClusterBIVc

MLTFSLMLVLFFEFMGAAISKICHVQKAQILSKHQQKALIEHELKRCFTNIKNQNDKNSSQCGQVWDGVMCWPPVEHGTTSVQQCPSYIHKFKPDGFATRICLENGDWFVHPDLNKTWTNYSGCVGNMTDNQSLVPDVIQEHMPRIQIFYNIGYGISLVSLLVAVTLMIYFRKLHCQRNTVHINMFISFILRSIICFIKDIDITPEVYISSTIEEETTFGQASICKSVYVIFYYVLTANFMWIFVEGLFLHTFVLSTKYNVSRRLYRTFLILGWCVPLLSVIPWVIVRYMFENTLCWNTMDNRFFWIIKGPIMVTCIINLVFFINIIRVLYTKLNAAHTKDPNKYRKLARSTLVLIPLFGVYYAVFIAVPICMDPKLEVIWMYSEMFFNSFQGFAVALLFCFMNGEVQREIKKHWRRRRIMRCQRNMSSRSKTFITDHDTHHTCIPDTWDTNGNMDLKELTIPVCNVDIDLNVNSPQELDENLPILKP

*Laternula eliptica*

>Lel.CALCR

LTCFRQLNCVRVTLHKHLFVSYVLTGAMWILYYRLVPMDPEVLMHNALWCRVLHVLTHYMTVCNYAWMFCEGFYLNAVIVITF

>Lel.ClusterB

PRTWDGMMCWPPTLRGTLASQPCPDYVHGFYTTGHSTKQCLDTGQWYYSPEFNNTWTNYTNCIRSPSPITN

*Pecten maximus*

>Pma.CALCR

MPGNDSLSKSNHLQDLLNASFLKCLREVLSQPYPNDGEQYCNATSDIFGCWNYTQAGTTAIIPCPEIPGMNPNEYAFKQCTENGTWWRSPSTNKEKSDYSYCWKDIHTFIENTNSGHEHVYIFVSGYSISVAMLLLSLFIFCRFSQLRCDRITIHKNLFSSYVLTGFSWILYMLLVATNGHVIQENPIWCRMIHVFAQYCVVSNFSWMFCEGLYLHTIMVRTFGSTKHVLIACYFIGWLWPFVLVSIYTGIRGSADDDQRSSCWIGESSLQWIMYAPIIVSMVANFIFLCNIVRLLITKLRQVPEATQTKKAARATLILIPLLGLQFLLVPLRPEIGSDTERAYHYISALVVSLQGAFVSTMYCFCNSEVISVIKRKWYQHKVMFSGKRSRTSSTIAATTYTFVDHVSTVQTSIT
